# Unravelling the taxonomy of an interstitial fish radiation: Three new species of *Gouania* (Teleostei: Gobiesocidae) from the Mediterranean Sea and redescriptions of *G. willdenowi* and *G. pigra*

**DOI:** 10.1111/jfb.14558

**Published:** 2020-11-03

**Authors:** Maximilian Wagner, Marcelo Kovačić, Stephan Koblmüller

**Affiliations:** ^1^ Institute of Biology University of Graz Graz Austria; ^2^ Department of Biology University of Antwerp Antwerp Belgium; ^3^ Natural History Museum Rijeka Rijeka Croatia

**Keywords:** blunt‐snouted clingfish, cryptobenthic fish, DNA‐barcoding, intertidal, pebble beach

## Abstract

The clingfish (Gobiesocidae) genus *Gouania* Nardo, 1833 is endemic to the Mediterranean Sea and inhabits, unlike any other vertebrate species in Europe, the harsh intertidal environment of gravel beaches. Following up on a previous phylogenetic study, we revise the diversity and taxonomy of this genus by analysing a comprehensive set of morphological (meristics, morphometrics, microcomputed tomography imaging), geographical and genetic (DNA‐barcoding) data. We provide descriptions of three new species, *G. adriatica* **sp. nov.**, *G. orientalis* **sp. nov.** and *G. hofrichteri* **sp. nov.**, as well as redescriptions of *G. willdenowi* (Risso, 1810) and *G. pigra* (Nardo, 1827) and assign neotypes for the latter two species. In addition to elucidating the complex taxonomic situation of *Gouania*, we discuss the potential of this enigmatic clingfish genus for further ecological, evolutionary and biodiversity studies that might unravel even more diversity in this unique Mediterranean fish radiation.

## 1 INTRODUCTION

Characterized by a cryptic lifestyle, a small size and a strong association to the benthos, cryptobenthic fishes are among the least studied marine vertebrates on this planet (Brandl *et al*., 2018). Their secretive ecology entails major problems for researchers collecting these fishes (Ackerman & Bellwood, 2000; Smith‐Vaniz *et al*., 2006), typically confining systematic and taxonomical studies to the investigation of only a few individuals (*e.g*., Hastings & Conway, 2017). Above all, many cryptobenthic taxa contain morphologically similar or, at least at first glance, identical species, such that their overall biodiversity is drastically underestimated, even in considerably well‐studied groups or geographic regions (*e.g*., Conway *et al*., 2014; Tornabene *et al*., 2015; Wagner *et al*., 2019; Winterbottom *et al*., 2014). However, the inclusion of molecular methods in classic taxonomic studies has proven to be particularly effective for overcoming these obstacles and in recent years genetic data have become a key tool for resolving phylogenetic relationships among and within cryptobenthic taxa (*e.g*., Almada *et al*., 2008; Conway *et al*., 2014; Henriques *et al*., 2002; Hoban & Williams, 2020; Kovačić *et al*., 2020; Tornabene *et al*., 2010; Victor, 2013; Winterbottom *et al*., 2014).

This is also true for the family of clingfishes (Gobiesocidae) that comprises around 180 species in 50 genera worldwide (Fricke *et al*., 2020a). Clingfishes possess a thoracic adhesive disc that enables them to tenaciously cling to even very slimy and rough surfaces (Ditsche & Summers, 2019; Wainwright *et al*., 2013). The ability to stick tight on different substrates can be considered as a prerequisite for invading empty ecological niches and is a comparatively energy‐efficient way to persist in a high energetic environment, such as the intertidal zone (Davison, 1985). Still, due to their cryptic ecology, it is not surprising that the majority of recent genus and species descriptions are based on the investigation of a few individuals only (*e.g*., Conway *et al*., 2017b). Whereas some of these taxonomical and systematic studies use more classical approaches (*e.g*., Fricke, 2014; Fricke *et al*., 2010, 2015; Fricke & Wirtz, 2017, 2018; Sparks & Gruber, 2012), many authors include more comprehensive morphological (*e.g*., micro‐computed tomography imaging) and/or genetic (*e.g*., single locus DNA barcoding) methods, which altogether turn out to be effective tools for delineating clingfish species (*e.g*., Almada *et al*., 2008; Bilecenoğlu *et al*., 2017; Conway *et al*., 2014, 2017a–c, 2018, 2019; Fricke *et al*., 2017; Fujiwara *et al*., 2018; Fujiwara & Motomura, 2018a,b, 2019, 2020; Henriques *et al*., 2002).

From the Mediterranean Sea, nine species in six genera have been described and almost all of them exclusively inhabit upper littoral ecosystems (Bilecenoğlu *et al*., 2017; Hofrichter & Patzner, 2000). One of them, *Gouania willdenowi* (Risso 1810), the only described species of the genus *Gouania* Nardo, 1833, has stenoeciously adapted to life in the interstitial of Mediterranean intertidal gravel beaches (Hofrichter & Patzner, 2000; Wagner *et al*., 2019). The narrow interstitial space of intertidal gravel beaches is characterized by extreme mechanical (shearing forces) and tidal stressors (*e.g*., daily desiccation) and can be considered as one of the most demanding environments for fishes (see Supporting Information Video S1 for behavioural observation). Indeed, among all known fishes, only *Gouania* and some Pacific gobies of the genus *Luciogobius* Gill, 1859 have evolved adaptations to live in this particular environment (Wagner *et al*., 2019; Yamada *et al*., 2009). *Gouania* is endemic to the Mediterranean Sea and, compared to other members of the subfamily Lepadogastrinae, is characterized by unique morphological modifications (*i.e*., increased number of vertebrae, small eyes, blunt snout) that can be linked to its interstitial lifestyle (Hofrichter, 1995; Wagner *et al*., 2019). These morphological adaptations include osteological changes of the median fins as well as the axial skeleton and become particular prominent if directly compared to the closely related sister genus *Lepadogaster* (Konstantinidis & Conway, 2010; Leray, 1961). Despite their high abundance in suitable habitats (up to 24 individuals/m^2^) (Hofrichter & Patzner, 2000), little is known about the ecology or biology of the species. Hofrichter (1995) and his subsequent work (Hofrichter & Patzner, 2000) were the first studies to focus on the habitat utilization of *G. willdenowi* and present important biological observations. For example, Hofrichter (1995) was the first person to document the spawning behaviour of *G. willdenowi* from Messina and rediscovered, after Facciolà (1887), a unique sexual dimorphism with males showing seemingly perfused finger‐like extensions on the edges of the sucking disc. More recently, studies showed that *Gouania* likely exhibits passive amphibious emergence behaviour, an essential prerequisite for survival in the intertidal zone (Bilecenoğlu, 2015; Hofrichter & Patzner, 2000).

Thus far, phylogenetic and population genetic studies about European clingfishes have been scarce and biased towards the genus *Lepadogaster* (*e.g*., Henriques *et al*., 2002; Klein *et al*., 2016; Wagner *et al*., 2017). Even though Hofrichter (1995) and Hofrichter and Patzner (2000) document striking colour differences between populations, the diversity within *G. willdenowi* has been overlooked for more than two decades. In a previous study that included *Gouania* from large parts of the Mediterranean Sea, we found two distinct morphotypes (“stout” and “slender”) that live in sympatry in the Adriatic and eastern Mediterranean basin, but five deeply divergent genetic lineages, implying repeated independent evolution of convergent phenotypes within the last ~5 million years (Wagner *et al*., 2019). The taxonomy of these genetically distinct lineages, however, remained unresolved. In general, the taxonomic history within the genus *Gouania* and all other Mediterranean clingfishes is complex and characterized by independent descriptions and synonymizations in the 19th century (Briggs, 1955; reviewed by Hofrichter, 1995). Above all, type material is lacking for *G. willdenowi* or for junior synonyms, such as *G. pigra*, from the previously investigated geographical range.

We provide an integrative taxonomic approach, through combining morphological, geographical and genetic data, aimed at clarifying the diversity and taxonomy of the intertidal clingfish genus *Gouania*. We deliver descriptions of three new species of the genus and redescriptions (including the designation of neotypes) for *G. willdenowi* and the now resurrected *G. pigra*. Furthermore, we not only elucidate the complex taxonomic situation of *Gouania*, but also discuss the potential for further ecological and evolutionary studies that could unravel even more diversity and open up thrilling questions regarding this enigmatic endemic Mediterranean fish radiation.

## 2 MATERIALS AND METHODS

### 2.1 Sampling and assessment of distribution ranges

**FIGURE 1:**
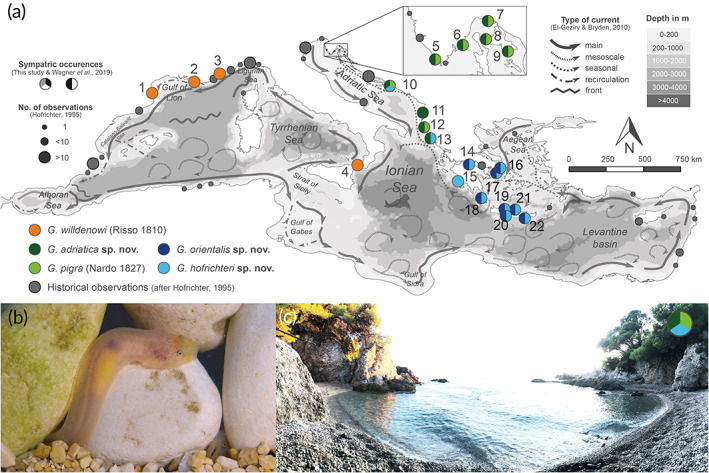
Geographical distribution ranges and ecology. (a) Distribution ranges of single *Gouania* species. The data shown are based on genetic, morphological and field observations as well as on historical findings mentioned by Hofrichter (1995) (all his records combined the species in *G. willdenowi* described here). Type and location of water currents are based on data provided by El‐Geziry and Bryden (2010). Numbers 1–23 indicate sampling sites (compare with Supporting Information Table S1). (b) Female *Gouania pigra* (Nardo, 1827) in the interstitial of pebbles (photo taken in aquarium); see Supporting Information Video S1 for behaviour. (c) Trstenik (Pelješac, Croatia) – a site where *G. pigra*, *G. adriatica* **sp. nov.** and *G. hofrichteri* **sp. nov.** were found in sympatry. Photographs by M. Wagner

Sampling was conducted from 2014 to 2019 at 22 sites across the Mediterranean Sea (see Figure 1a and Supporting Information Table S1). Specimens were collected on intertidal pebble beaches by pulling a bucket through the gravel, and in boulder fields between 0 and 1 m depth by lifting the stones and catching the fish using aquarium nets. The fish were euthanized with an overdose of MS‐222.

Species distribution ranges were assessed using already published (Wagner *et al*., 2019) and newly generated DNA‐barcode sequences, as well as by morphological investigation of nontype specimens deposited in the Natural History Museum Rijeka, Rijeka, Croatia (for voucher numbers see Supporting Information Table S2). Additionally, historical data on distribution and number of observations of *G. willdenowi* were included in the analysis from records summarized by Hofrichter (1995).

**Ethical statement.** Fish collection and euthanasia was carried out with the approval of the Ethics Committee of the University of Graz (permit No. GZ. 39/54/63 ex 2019/20), in accordance with the EU‐Directive 2010/63/EU, Annex IV and the Austrian Animal Experimentation Ordinance, §20.

### 2.2 Morphological investigation

Morphometric methods, measurements and definitions are modified from Briggs (1955) to match the unique morphology of *Gouania* with reduced dorsal and anal fins and the lack of a caudal peduncle. Standard length (SL) is measured from the median anterior point of the upper lip to the base of the caudal fin (posterior end of the hypural plate). Measurement data are given as a ratio in the text and as percentages of SL in Table 1. Other measurements in alphabetic order: body depth at anus is vertical distance from anus to dorsal edge of body; body depth at pectoral fins is vertical distance at pectoral‐fin origin from the bottom of the ventral disc to the dorsal edge of the body; body width at anus is body width at anus in ventral view; body width at pectoral fins is maximum body width at pectoral‐fin origin including the pectoral fin origin in dorsal view; caudal base depth at origin of caudal fin is the vertical distance from the dorsal edge of the body to the ventral edge of the body at the vertical through the midpoint of origin of the caudal fin; caudal‐fin length is the distance from the base of the caudal fin at the midpoint (posterior end of the hypural plate) to the most distant ray tip; disc length is the longitudinal distance between the outermost edges of the thin membrane surrounding heavier portions of the disc in ventral view; disc width is the maximum disc width between the tips of the ventral rays in ventral view; distance between the posterior margin of sucking disc and anus is the distance between the outermost edge of the posterior thin membrane of the disc and the anus in ventral view; head depth at anterior sucking disc edge is the vertical distance from the dorsal edge of the head to the ventral edge of the head at the anterior sucking disc edge; head depth at orbit is the vertical distance from the dorsal edge of the head to the ventral edge of the head at mideye; head length is the distance from the median most anterior point of the upper lip to the most posterior part of the opercular edge in lateral view; head width at head invagination is the maximum body width at head invagination in dorsal view; head width at orbit is the maximum body width at mideye in dorsal view; head width at sucking disc anterior edge is the maximum body width at the sucking disc anterior edge in ventral view; horizontal eye diameter is the maximum horizontal length of the externally visible eye; interorbital distance is the smallest distance between visible eyes; pectoral‐fin length is the length of the longest ray of the pectoral fin from origin to tip in lateral view; postanus length is the distance from the vertical of the anus to the midpoint of the base of the caudal fin (posterior end of the hypural plate) in lateral view; postorbital distance is the distance from the posterior edge of the eye to the upper opercular posterior edge in lateral view; preanus length is the distance from the median most anterior portion of the lower lip to the anus in ventral view; predisc length is the distance from the median most anterior portion of the lower lip to the anterior membranous edge of the disc in ventral view; preorbital distance is the distance from the median most anterior point of the upper lip to the anterior edge of the eye in dorsal view; prepectoral distance is the distance from the median most anterior point of the upper lip to the upper edge of the pectoral‐fin base in lateral view; vertical eye diameter is the maximum vertical length of the externally visible eye. Measurements smaller than 20 mm were taken with interactively selected points in Olympus cellSens Entry 2.2. software using an Olympus SC180 camera with an Olympus U‐TV0.5XC‐3 adapter on the stereomicroscope Olympus SZX10, while those out of this range were taken by a digital calliper. Fin ray counts follow Briggs (1955) with the caudal principal rays being those with noticeably free tips. The terminology of the complex system of cephalic superficial neuromast rows in *Gouania* was expanded from Conway *et al*.'s (2017a) terminology for the reduced system of rows in *Trachelochismus*: supralabial row (SR), located medially above the upper lip; nasal row (NR), located dorsal to nostrils; longitudinal infralateral row (LIR), located dorsal to the lateral part of the upper lip, along the lower cheek to preopercle, discontinuous at the vertical level of the anterior ventral and suborbital transversal rows; suborbital transversal row (STR), located on the lateral side of the head ventral to the orbit; postorbital transversal row (POR), located posterior and ventral to the posterior margin of the orbit; preopercular transversal row (PTR), located on the skin covering the preopercle; subopercular longitudinal row (SLR), located along the skin covering the subopercle; mandibular row (MR), located on the ventral surface of the lower jaw behind the lower lip; anterior ventral row (AVR), located posterior and ventral to the posterior angle of the jaws; posterior ventral row (PVR), located on the skin ventral to the preopercle; anterior dorsal row (ADR) located on the nape behind the interorbital region; posterior dorsal row (PDR), located medially on the nape behind the anterior dorsal row and distantly from the interorbital region; hyomandibular row (HR), located on the skin dorsal to the preopercle; supraopercular row (SR1), located on the skin dorsal to the posterior opercular edge; suprapectoral row (SR2), located dorsal to the upper edge of the pectoral‐fin base; dorsolateral longitudinal row (DLR), located on the upper lateral side of the body; ventrolateral longitudinal row (VLR), located on the lower lateral side of the body. The cephalic sensory pore terminology follows that of Shiogaki and Dotsu (1983). Granules are small dermal structures visible as small bumps on the skin surface. Adhesive disc papillae terminology follows Briggs' (1955) disc regions: A, the anterior part; B, the posterior part; C, the central part. The type material was reversibly stained in a 2% solution of Cyanine Blue in distilled water following the method of Saruwatari *et al*. (1997) and with our specific protocol: the specimens were briefly dried in the air and then kept for 60 s in the staining solution. After examination they were stored in 70% ethanol where they reached the original state, *i.e*., completely lost any trace of staining. The preservative with diluted stain from the specimens was replaced with fresh ethanol 24 h after staining.

**TABLE 1:** Morphometric characters (% SL) of *Gouania* described/redescribed species

Species	*G. adriatica* sp. nov.		*G. orientalis* sp. nov.		*G. hofrichteri* sp. nov.		*G. pigra* (Nardo, 1827)		*G. willdenowi* (Risso, 1810)		
Specimen	Holotype	Paratypes	Holotype	Paratypes	Holotype	Paratypes	Neotype	Other material	Neotype	Other material
Sex	Male	Males and females	Male	Males and females	Male	Males and females	Female	Males and females	Male	Males and females
Number of specimens	1	9	1	9	1	9	1	9	1	9
Standard length (SL) in mm	41.41	22.95–41.03	32.8	17.09–37.53	30.35	20.62–36.91	43.12	32.58–39.48	46.11	31.13–46.11
% standard length										
Body depth at anus	15.3	12.9–16.2	14.0	12.7–15.2	11.0	9.1–11.7	12.3	11.3–13.5	11.3	12.5–13.8
Body depth at pectoral fins	15.7	11.2–14.8	13.0	11.6–14.4	10.1	9.5–10.9	11.5	10.6–11.8	12.2	11.4–13.4
Body width at anus	12.1	9.8–13.0	11.4	10.8–12.8	8.6	7.4–9.1	9.9	8.5–11.4	8.8	9.6–11.3
Body width at pectoral fins	16.8	13.7–16.6	14.5	14.2–15.7	12.3	10.3–12.4	11.8	11.5–13.2	12.9	13.9–15.8
Caudal base depth	12.6	10.1–13.5	13.0	11.4–14.1	9.2	8.2–10.4	9.5	9.0–10.8	11.3	11.5–13.0
Caudal fin length	16.3	13.9–16.4	15.0	14.2–17.5	12.1	11.5–13	11.7	11.1–12.7	14.2	13.5–15.5
Disc length	20.1	15.9–19.0	17.3	14.9–19.0	12.1	10.2–13.4	13.5	12.1–14.6	17.5	17.1–19.3
Disc width	22.3	16.3–19.0	18.5	16–18.2	15.0	12.4–16.1	14.7	13.5–18.5	19.9	17.4–20.2
Distance between the posterior margin of sucking disc and anus	25.1	22.7–26.1	21.9	20.2–24.2	28.0	26–29.6	31.3	25.7–34.7	20.8	20.2–23.7
Head depth at anterior sucking disc edge	14.1	11.1–14.3	13.5	12.1–14.9	11.4	9.9–12.2	10.9	11.3–12.8	13.3	11.3–14.0
Head depth at orbit	12	8.8–12.2	10.0	9.4–10.8	8.7	7.3–9.3	8.5	8.0–9.8	11.0	9.4–12.4
Head length	30	26.2–29.4	28.8	25.0–28.9	21.6	18.9–23.4	19.5	20.1–22.9	26.8	24.3–28.8
Head width at head invagination	19.4	15.6–20.4	17.3	15.0–17.8	14.1	11.2–14.3	12.5	12.6–15.3	17.7	16.1–19.0
Head width at orbit	20.1	15.8–19.6	18.9	15.8–18.4	14.1	11.2–14.4	12.5	13.7–15.9	17.8	16.1–19.5
Head width at sucking disc anterior edge	20.6	17.7–23.7	20.4	18.3–21.3	16.0	14.1–17.3	15.6	14.2–18.4	19.6	19.0–21.9
Horizontal eye diameter	3.2	3.0–3.7	2.9	2.8–4.2	2.2	2.1–2.6	2.0	2.0–2.4	2.5	2.3–2.9
Interorbital distance	9.2	6.1–9.3	8.0	5.9–8.7	6.2	5.1–6.3	6.5	5.6–7.3	8.8	7.1–8.6
Pectoral fin length	10.7	8.7–11.3	9.0	8.2–10.3	5.9	5.4–6.4	5.8	5.6–7.5	9.2	8.6–9.3
Postanus length	41.3	38.1–42.2	43.5	35.5–43.9	43.4	41.8–52.6	42.5	42.4–46.6	39.5	39.9–42.3
Postorbital distance	18.5	15.1–18.3	17.5	15.2–18.1	13.4	12–14.5	14.0	12.6–15.2	15.9	15.1–17.2
Preanus length	62.3	57.7–63.5	60.1	55.9–60.8	55.5	50.8–57.1	56.1	52.8–59.4	54.1	55.8–60.8
Predisc length	18.6	17.1–19.7	19.8	15.9–19.4	15.3	14.2–16.5	15.1	13.2–16.5	16.9	16.4–18.4
Preorbital distance	9.4	7.5–9.7	9.0	7.2–9.1	7.2	5.7–7.8	7.1	5.5–8.0	9.5	8.3–10.0
Prepectoral‐fin length	28.2	25.1–28.5	27.8	24.7–28.2	22.1	19.5–23.7	22.2	19.5–23.4	26.6	24.4–28.4
Vertical eye diameter	4.3	3.4–4.1	3.0	2.5–4.3	2.6	2.0–2.7	2.9	2.3–2.9	3.3	2.6–3.1

*Note*: Values for each species in the table presented as holotype/neotype and separately the range for species paratypes/other material. Characters are sorted in alphabetic order.

Osteology was investigated on four to six specimens per species based on three‐dimensional (3D) models from microcomputed tomography (microCT) images using a MicroCT 40 device (SCANCO Medical, Wayne, PA, U.S.A.) with a resolution of 15 or 20 μm. 3D modelling was conducted in Drishti v.2.6.4 (Limaye, 2012) and the osteological terminology follows Springer & Frase (1976). Throughout the text we refer to “caniniforms” as teeth having a conical, elongated and recurved shape. All the microCT data are from Wagner *et al*. (2019) and therefore not all scans represent type material (mainly due to bad fixation). However, all investigated structures were verified with nontype material where scans were available (see Supporting Information Table S2 for more information).

All the investigated vouchers and the type material have been deposited in the Natural History Museum, Rijeka, Croatia (PMR) and at the Zoological State Museum, Munich, Germany (ZSM).

### 2.3 Molecular genetics and comparative methods

Previously published cytochrome‐c oxidase subunit I sequences (COI barcodes) of *Gouania* and *Lepadogaster* specimens were downloaded from GenBank (accession numbers MK873443–MK873539, MF425774, MF425776–MF425781, MF544114–F544117). All these sequences were produced in the framework of Wagner *et al*. (2017, 2019). Additionally, COI barcodes were generated for 27 further individuals (see Supporting Information Table S2), using the primers FishF1 (5′ TCAACCAACCACAAAGACATTGGCAC 3′) and FishR1 (5′ TAGACTTCTGGGTGGCCAAAGAATCA 3′) designed by Ward *et al*. (2005). Procedures of amplification and sequencing follow the protocols in Koblmüller *et al*. (2011) and Wagner *et al*. (2019). Sequences were aligned using MUSCLE (Edgar, 2004) and calculations of net interspecific evolutionary divergence between groups, using 1000 bootstraps replications and the Kimura 2‐parameter model, were conducted in MEGA v.07 (Kumar *et al*., 2016). For estimating the “barcoding gap” (Hebert *et al*., 2003) the minimum interspecific and the maximum intraspecific divergence (in %) were calculated in R vs. 3.6.0 (R Core Team, 2017) using the functions “nonConDist” and “maxInDist” from the R‐package SPIDER (Brown *et al*., 2012). The phylogenetic relationships between single *Gouania* lineages are visualized based on a schematic (multilocus) multispecies coalescent tree inferred by Wagner *et al*. (2019). All newly generated COI sequence data were deposited on GenBank under accession numbers MT299844–MT299870 (Supporting Information Table S2).

## 3 RESULTS

### 3.1 Taxonomy

#### 3.1.1 *Gouania* Nardo, 1833

*Gouania* Nardo 1833: 548 (type species: *Gouania prototypus* Nardo 1833 by original designation. Genus appeared first as *Covania*, name corrected by Canestrini, 1864:181).

**Diagnosis.** The genus diagnosis is based on the genus description by Briggs (1955) and adjusted to fit the new species: dorsal and anal fins reduced to low ridges with very weak rays, connected to the caudal fin. Ventral adhesive disc of „double„ type, with no papillae in region A and flattened papillae in regions B and C. Disc small, 5.0–9.8 in standard length. Body slender and elongated, posteriorly laterally compressed. Head rounded in dorsal outline; snout not produced. Upper jaw with outer row of medium‐sized caniniforms frontally. Behind them irregularly scattered small conical inner teeth. Outer row continues laterally as large caniniforms, followed behind by medium‐sized caniniforms. Lower jaw with outer row of medium‐sized caniniforms frontally. Behind them irregularly scattered small conical inner teeth. The single row of larger caniniforms continuous laterally. Vertebrae 35–40. The first gill arch with hemibranch, the 2nd to 4th gill arches with holobranchs. No fleshy pad present on lower pectoral base. No subopercular spine. Gill membranes attached to isthmus. Six branchiostegals.

**FIGURE 2:**
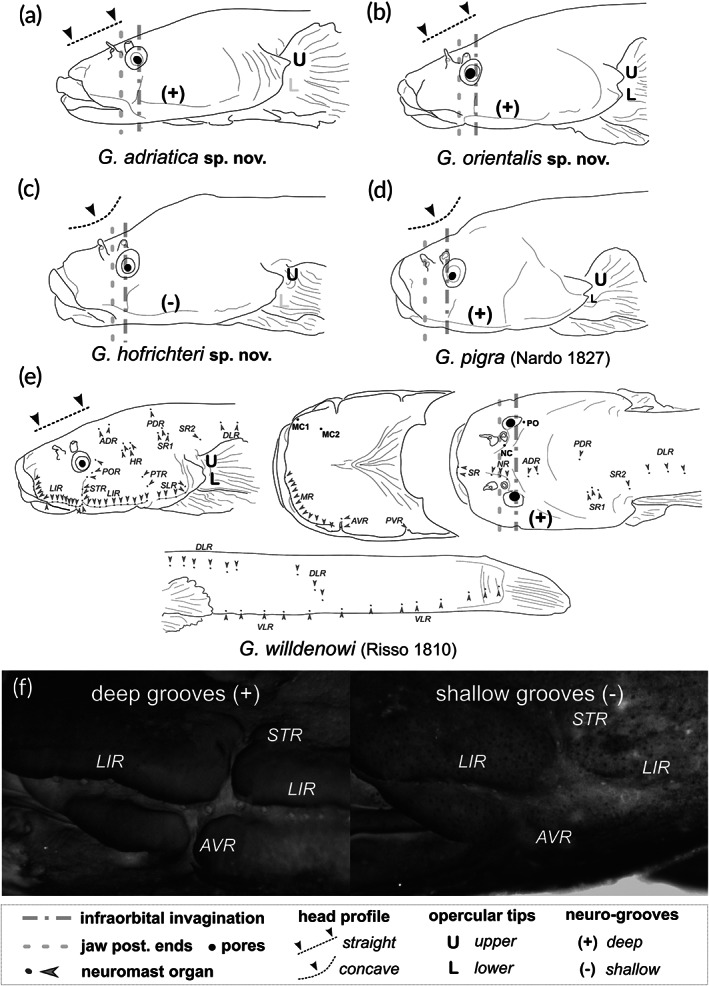
Comparative morphological overview and lateral line system. (a) Main morphological characteristics of head region of (a) *G. adriatica* **sp. nov.** (PMR VP4618 – Holotype), (b) *G. orientalis* **sp. nov.** (PMR VP4585 – Holotype), (c) *G. hofrichteri* **sp. nov.** (PMR VP4595 – Holotype) and (d) *G. pigra* (Nardo 1827) (PMR VP3529 – Neotype). (e) Position of pores (bold) and neuromasts (italic) shown on the example of *G. willdenowi* (Risso 1810) (PMR VP4574 – Neotype) and the main morphological characteristics in the head region of this species. (f) Longitudinal infralateral and suborbital transversal rows of superficial neuromasts can be placed on the well‐defined bottom of a deep (+) or shallow (–) groove. U, upper opercular tip; Abbreviations: L, lower opercular tip; SR, supralabial row; NR, nasal row; LIR, longitudinal infralateral; STR, suborbital transversal row; POR, postorbital transversal row; PTR, preopercular transversal row; SLR, subopercular longitudinal row; MR, mandibular row; AVR, anterior ventral row; PVR, posterior ventral row; ADR, anterior dorsal row; PDR, posterior dorsal row; HR, hyomandibular row; SR1, supraopercular row; SR2, suprapectoral row; DLR, dorsolateral longitudinal row; VLR, ventrolateral longitudinal row. Photographs by M. Wagner and M. Kovačić

**Key to the species of *Gouania*** (Figure 2).

1a. Dorsal head profile concave above eye (Figure 2c,d), caudal‐fin length 11.1–13.0% of standard length, pectoral‐fin length 5.4–7.5% of standard length, “slender” species of *Gouania* … 2

1b. Dorsal head profile straight above eye (Figure 2a,b,e), caudal‐fin length 13.5–17.5% of standard length, pectoral‐fin length 8.2–11.3% of standard length, “stout” species of *Gouania* … 3

2a. Posterior angle of jaws extends to, or close to, a vertical line drawn through the anterior edge of the anterior nostril; longitudinal infralateral and suborbital transversal rows of superficial neuromasts placed in the well‐defined deep groove (Figure 2d,f); Adriatic Sea … *Gouania pigra*

2b. Posterior angle of jaws extends to between a vertical line drawn through posterior edge of anterior nostril and a vertical line drawn through anterior edge of eye; longitudinal infralateral and suborbital transversal rows of superficial neuromasts placed in shallow groove disappearing in posterior part of longitudinal infralateral row (Figure 2c,f); Aegean Sea, rare in the south Adriatic Sea (a single record from Pelješac, Croatia) … *Gouania hofrichteri* **sp. nov.**

3a. Posterior opercular edge with pointed upper tip and rounded lower posterior edge (Figure 2a); Adriatic Sea, northern Ionian Sea (Island of Corfu) … *Gouania adriatica* **sp. nov.**

3b. Posterior opercular edge with two equally long tips (Figure 2b,e) … 4

4a. Vertebrae 37–38; West Mediterranean to Messina … *Gouania willdenowi*

4b. Vertebrae 35–36; southern Ionian Sea and Aegean Sea … *Gouania orientalis* **sp. nov.**

#### 3.1.2 *Gouania adriatica* sp. nov.

**English name: Adriatic blunt‐snouted clingfish**

*ZooBank LSID*: urn:lsid:zoobank.org:act:F054E7C9‐604F‐41DA‐8068‐145DDCCC1FBE

**FIGURE 3:**
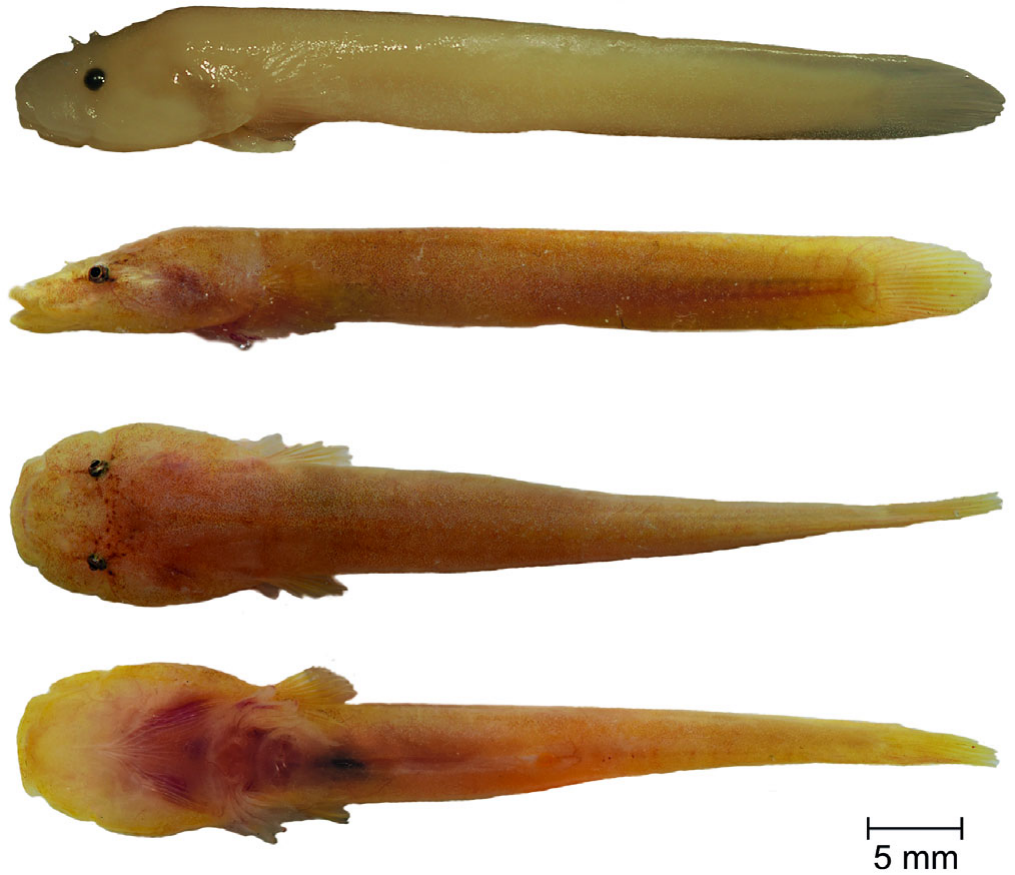
*Gouania adriatica* **sp. nov.,** PMR VP4618, holotype, male, 41.41+6.77 mm, Stoja, Pula. Lateral view of specimen preserved in 4% formaldehyde (top). Lateral, dorsal and ventral view, alive (below). Photographs by M. Wagner and M. Kovačić

**Holotype**. PMR VP4618, male, 41.41 + 6.77 mm, Stoja, Pula, Croatia, 44°51′38.4″N, 13°49′05.0″E, coll. M. Wagner, July 17, 2016 (Figure 3).

**Paratypes**. ZSM‐PIS‐047650, male, 41.03 + 5.89 mm Envi beach, Vlorë, Albania, 40°23′16.3″N, 19°28′58.2″E, coll. M. Wagner, August 14, 2019; ZSM‐PIS‐047652, male, 27.3 + 4.27 mm, Trstenik Pelješac, Croatia, 42°54′07.7″N, 17°25′47.0″E, coll. M. Wagner, August 18, 2019; ZSM‐PIS‐047651, male, 34.53 + 5.65 mm, Stara Baška, Krk, Croatia, 44°56′45.3″N, 14°42′22.2″E, coll. M. Wagner, September 16, 2019; PMR VP3523, juvenile of unidentified sex, 22.95 + 3.6 mm, Glavotok, Krk, Croatia, 45°05′44.9″N, 14°26′32.4″E, coll. M. Wagner, May 13, 2015; PMR VP3524, male. 26.19 mm, caudal fin damaged and PMR VP3525, female, 28.4 + 4.23 mm, both from Glavotok, Krk, Croatia, 45°05′44.9″N, 14°26′32.4″E, coll. M. Wagner, May 15, 2015; ZSM‐PIS‐047653, female, 34.42 + 4.85 mm and ZSM‐PIS‐047653, female, 35.23 + 4.9 mm, both from Pećine, Rijeka, Croatia, 45°18′52.4″N, 14°28′11.7″E, coll. M. Wagner, July 12, 2019; PMR VP4580, female, 34.7 + 4.99 mm, Sv. Marina, Istria, Croatia, 45°01′42.1″N, 14°09′17.4″E, coll. M. Wagner, July 18, 2015.

**Diagnosis**. *Gouania adriatica* **sp. nov.** differs from its congeners by the combination of the following characters: (1) dorsal head profile a straight line from nape above eye to upper lip tip; (2) posterior angle of jaws extends to between a vertical line drawn through posterior edge of anterior nostril and a vertical line drawn through anterior edge of eye; (3) pointed upper and rounded lower posterior opercular edge; (4) longitudinal infralateral and suborbital transversal rows of superficial neuromasts placed in the well‐defined deep groove; (5) body cross‐section behind pectoral fin base half oval with straight ventral side; (6) the granules on body shallow and inconspicuous; (7) upper attachment of gill membrane opposite to 5th to 6th pectoral ray; (8) principal caudal‐fin rays 12–13; (9) vertical eye diameter 3.4–4.3% of standard length; (10) horizontal eye diameter 3.0–3.7% of standard length; (11) head length 26.2–30.0% of standard length; (12) pectoral‐fin length 8.7–11.3% of standard length; (13) prepectoral distance 25.1–28.5% of standard length; (14) ventral adhesive disc length 15.9–20.1% of standard length; (15) caudal‐fin length 13.9%–16.4 of standard length; (16) low number of vertebrae (= 35); (17) pharyngeal jaws with ceratobranchial 5 small, having several (about 5) small, conical teeth; (18) nasal bones club‐shaped; (19) star‐like pigmentation around eyes, reduced body pigmentation with no visible stripes.

**Description**. *General morphology*: Body proportions are given in Table 1. Body slender and elongated, posteriorly laterally compressed, body depth at pectoral fins 6.4–8.9 in SL, body depth at anus 7.7–10.2 in SL, body depth in width at pectoral fins 1.1–1.4, body depth in width at anus 0.7–0.8. Body cross‐section behind pectoral fin base half oval with straight ventral side. Granules on body shallow and inconspicuous, making skin surface more dotted than granulose. Head dorsoventrally compressed, head depth in width at orbit 1.5–1.8, and moderately large, head length 3.3–3.8 in SL, head wider than body width maximum, head width at anterior sucking disc edge 0.7–0.9 in body width at pectoral fins. Dorsal head profile a straight line from nape above eye to upper lip tip. Head rounded in dorsal view. Snout large compared to eyes, preorbital distance 2.9–3.7 in head length, 0.3–0.5 in horizontal eye diameter. Snout wide, not produced, blunt. Internostril space gently convex. Eyes dorsolateral, with lower eye edge rounded. Eyes small, 7.3–9.5 in head length, vertical diameter of the eye 0.8–1.1 in horizontal eye diameter. Infraorbital invagination vertical to posterior part of eye or to mideye. Interorbital distance wide, 0.3–0.6 in horizontal eye diameter. Centre of eye much closer to tip of snout than to posterior margin of operculum, preorbital distance in postorbital distance 1.8–2.2. Anterior and posterior nostrils long tubes of about equal length. Nostrils well separated and posterior nostril located behind and dorsally to the anterior edge of eyes. Single large lobe at the posterior margin of anterior nostril or bilobed, longer than nostril. Posterior nostril rim crenate with no extension. Head lateral line system with canals with pores and with superficial neuromasts arranged in rows. Head canals reduced and pores small. Single pore in nasal canal near posterior nostril. Single pore in postorbital canal close to posterior eye edge. Two pores in mandibular canal, anterior one close to anterolateral angle of mouth, posterior pore slightly in front of vertical of posterior angle of jaws, posterior pore usually more prominent. Lachrymal as well as preopercular canals and pores absent. Rows of superficial neuromasts as follows: SR 2, NR 3, LIR 24–27, STR 1–3, POR 3–4, PTR 2, SLR 4–5, MR 9–11, AVR 2, PVR 1, ADR 2, PDR 1, HR 3, SR1 3, SR2 2, DLR 6–8, VLR 10–12. STR and LIR rows of superficial neuromasts placed in a well‐defined deep groove. DLR row of superficial neuromasts anteriorly starts above pectoral fin, continuous dorsolateral and ends posteriorly downwards at midlateral level above anus or behind it. VLR anteriorly starts behind pectoral fin base, continuous ventrolateral and ends posteriorly upwards with last papilla nearly at midlateral level at caudal fin base. Mouth terminal, upper and lower lips end about equally, lips fleshy, upper lip larger than the lower lip. Posterior angle of jaws extends to between vertical line drawn through posterior edge of anterior nostril and vertical line drawn through anterior edge of eye. Chin with bilobed or slightly bilobed fold at anterior edge covering MR row of superficial neuromasts. Gill membrane attached to isthmus, gill opening starting at the base of pectoral fin, with upper attachment of gill membrane opposite to 5th to 6th pectoral ray. Pointed upper and rounded lower posterior opercular edge. No subopercular spine. No fleshy pad present on lower pectoral base. Urogenital papilla present. Preanus length in postanus length 0.6–0.7. Anal papillae absent, area around anus only wrinkled.

**FIGURE 4:**
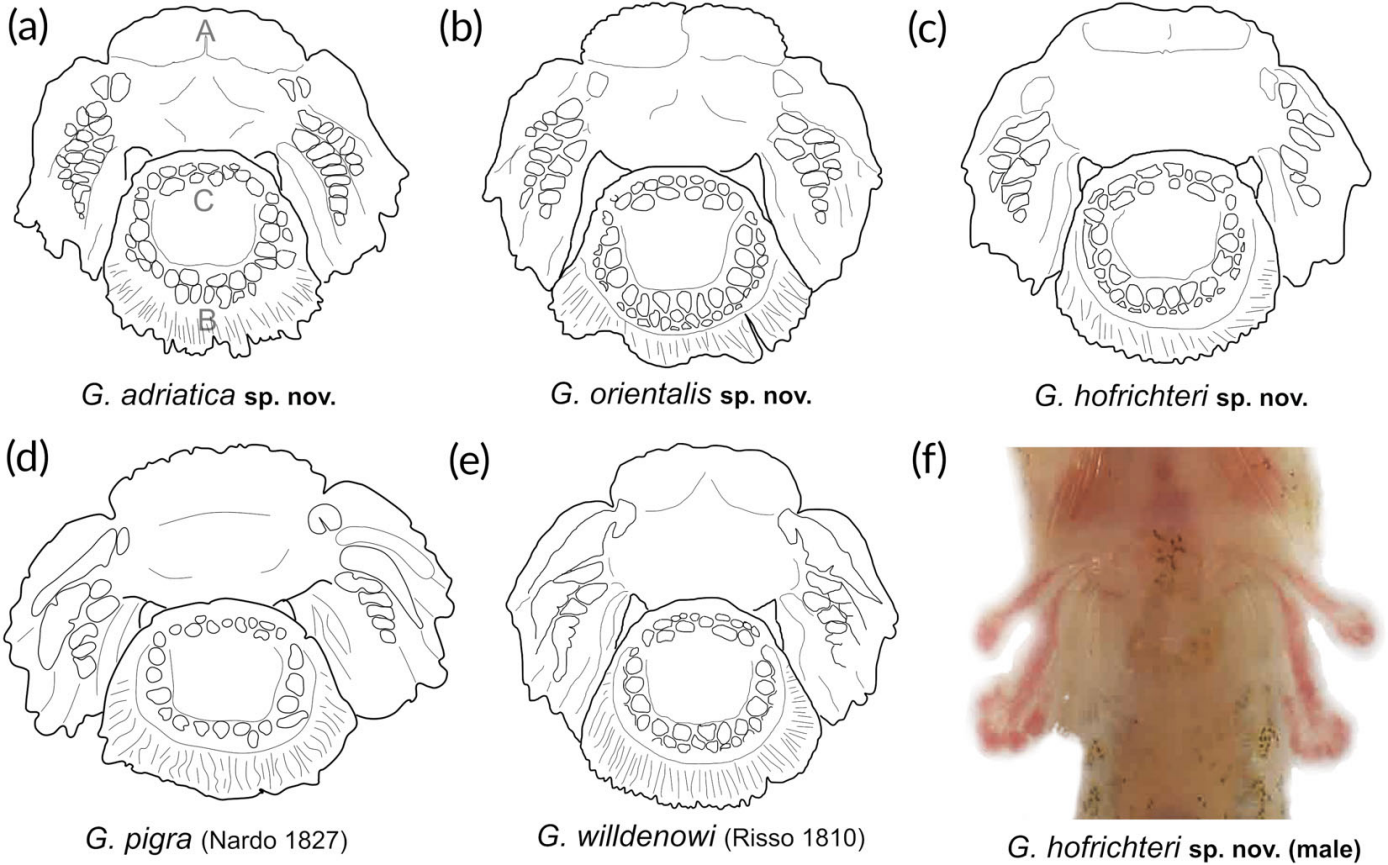
Sucking discs and disc‐papillae of *Gouania* species. (a) *G. adriatica* **sp. nov.** (PMR VP4618 – Holotype), (b) *G. orientalis* **sp. nov.** (PMR VP4585 – Holotype), (c) *G. hofrichteri* **sp. nov.** (PMR VP4595 – Holotype), (d) *G. pigra* (Nardo 1827) (ZSM‐PIS‐047649 – Other material) and (e) *G. willdenowi* (Risso 1810) (PMR VP4574 – Neotype). (f) Males of *Gouania* can have seemingly perfused prominent finger‐like extensions on sucking disc edge in region A.A, B and C correspond to disc regions. Photographs by M. Wagner

*Fins*. Rudimentary dorsal and anal fins located well posteriorly and short, reduced to low ridges with very weak rays, connected to caudal fin. Pectoral rays 15–17. Caudal fin rounded, principal caudal rays 12–13. Ventral adhesive disc (Figure 4a) of “double” type, anterior margin crenate with large invagination on each lateral side and in some specimen central invagination at midventral visible; posterior margin crenate or villous. Disc small, disc length 5.0–6.3 in SL, its width slightly larger than its length, width in length 0.9–1.0. No papillae in region A and flattened papillae in regions B and C. In region B one or two rows of papillae with total papillae count 10–30 and in region C two rows of papillae with total papillae count 9–15. No inner row of papillae on lateral sides of the central part of the anterior disc. Upper attachment of disc membrane attaching to base of pectoral fin at 15th–17th pectoral ray, *i.e*., at the ultimate or penultimate ray. In males, parts of disc region A appear to be perfused (see Figure 3, ventral view).

*Colouration*. Background colouration in life flesh‐coloured to yellow, slightly transparent and head pigmentation prominent, with a star‐like pattern around eyes (Figure 3). Body without (especially in juveniles) or with irregular melanocytes that are decreasing in density towards the posterior part of body or dotted (*e.g*., specimens from Vlorë) in life. Formaldehyde fixed specimens white to yellow and without pigments. In ethanol yellow or skin‐coloured with pigments still present. For more pictures of life colouration see Supporting Information File S1.

**FIGURE 5:**
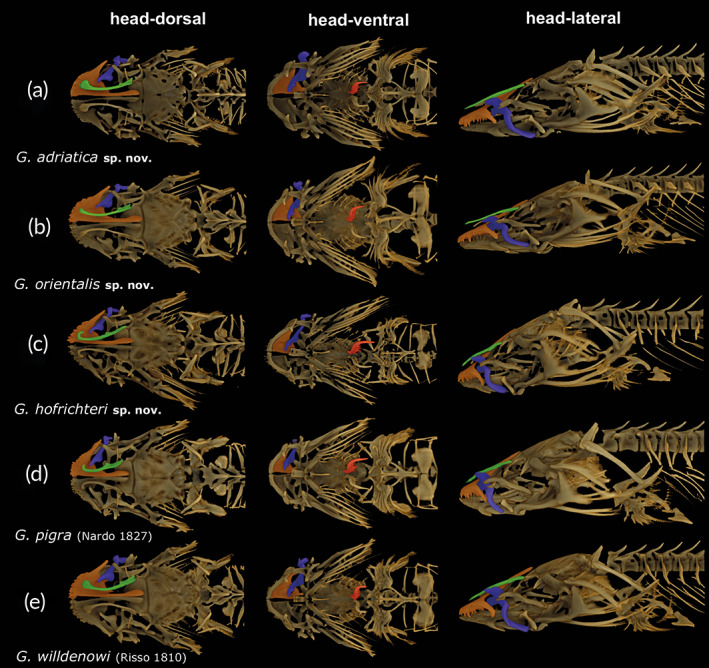
Head osteology of *Gouania* species. (a) *G. adriatica* **sp. nov.** (PMR VP4618 – Holotype), (b) *G. orientalis* **sp. nov.** (PMR VP4585 – Holotype), (c) *G. hofrichteri* **sp. nov.** (ZSM‐PIS‐ 047656 – Paratype), (d) *G. pigra* (Nardo 1827) (PMR VP3531 – Other material) and (e) *G. willdenowi* (Risso 1810) (ZSM‐PIS‐0476654 – Other material). Red, ceratobranchial 5; orange, premaxillary bone; blue, maxillary bone; green, nasal bone

*Dentition and osteology*. Upper jaw with outer row of about eight (one side) medium‐sized caniniforms frontally. Behind them inner small conical teeth irregularly scattered in two separate (left and right) drop‐like patches medially wide about five teeth, becoming narrowed to a single row of teeth laterally. Outer row continues laterally as two large caniniforms, followed behind by four or five medium‐sized caniniforms. Lower jaw with outer row of about 15 (one side) medium‐sized caniniforms frontally. Behind them single broad patch of small conical inner teeth medially wide about 5–6 teeth, becoming narrowed to a single row of teeth laterally. The single row of about eight larger caniniforms continuous laterally. Pharyngeal jaws with small ceratobranchial 5, having several (about 5) small, conical teeth (Figure 5a), pharyngobranchial 3 toothplate not visible on 3D models from microCT images. Number of vertebrae 35, abdominal 15 and caudal 20. The first gill arch with hemibranch, the 2nd to 4th gill arches with holobranchs. Subopercle indistinguishable from opercle, shaped as its posterior elongated extension, not forming or having subopercular spine. Six branchiostegals. Maxillary, premaxillary, nasal and ceratobranchial 5 bones shaped as in Figure 5a. Nasal bones club‐shaped.

**Etymology.** Named *adriatica*, meaning belonging to the Adriatic Sea, “Mare Adriaticum” or “Mare Hadriaticum” in Latin, which is the type locality of this species. The name is an adjective in the nominative singular (Article 11.9.1.1., ICZN, 1999).

**Ecology and geographical distribution (Figure** **1** **a).** The geographic distribution ranges from the northern Adriatic Sea to the northern Ionian Sea (Island Corfu). In the Adriatic basin, the species is broadly sympatric with *G. pigra*. Quantitative data on ecology is largely lacking. At one site in the Adriatic basin (Pelješac) it was found not just in sympatry, but even syntopy, also with *G. hofrichteri* **sp. nov.**, *i.e*., with both species in the same habitat (Figure 1c). Fish were found in the intertidal and sometimes syntopic with *Lepadogaster lepagaster*. During extreme low tide (late winter and early spring tides are the most extreme) this species was also found in layers of pebbles above the waterline.

**Remarks.** *Gouania adriatica* **sp. nov.** differs from slender‐bodied *Gouania* species (*G. pigra* and *G. hofrichteri* **sp. nov.**) by a dorsal head profile forming a straight line between nape above eye and upper lip tip (*vs*. dorsal head profile in lateral view “S” curved, concave above eye and convex at nape in slender‐bodied *Gouania* species), by a low number of vertebrae (Supporting Information Table S2; 35 *vs*. 38–40) and in life a star‐like pigmentation around eyes (*vs*. no star‐like pigmentation around eyes). Ten morphometric characters, as percentages of standard length, of *G. adriatica* **sp. nov** are nonoverlapping in range with both slender‐bodied *Gouania*: head length, head width at head invagination, vertical and horizontal eye diameter, body width at pectoral fins, pectoral‐fin length, prepectoral distance, ventral adhesive disc length, predisc length and caudal‐fin length (values in the Table 1). There are also morphometric characters nonoverlapping in range with only one of the two slender‐bodied *Gouania* (Table 1). In addition, *G. adriatica* **sp. nov.** differs from *G. pigra* by the posterior angle of jaws extending to between a vertical line drawn through the posterior edge of the anterior nostril and a vertical line drawn through the anterior edge of the eye (*vs*. posterior angle of jaws extending to, or close to, a vertical line drawn through the anterior edge of the anterior nostril) and principal caudal‐fin rays 12–13 (*vs*. principal caudal rays 10–11). *G. adriatica* **sp. nov.** also differs from *G. hofrichteri* **sp. nov.** by longitudinal infralateral and suborbital transversal rows of superficial neuromasts placed in a well‐defined deep groove (*vs*. longitudinal infralateral and suborbital transversal rows of superficial neuromasts placed in shallow groove disappearing in posterior part of longitudinal infralateral row), body cross‐section behind pectoral fin base half oval with straight ventral side (*vs*. body cross‐section behind pectoral fin base triangular with ventral flat and dorsal pointed), upper attachment of gill membrane opposite to 5th to 6th pectoral ray (*vs*. opposite to 3rd–4th pectoral ray), the granules on body shallow and inconspicuous (*vs*. granules on body, at least on posterior part and nape, large and prominent). *G. adriatica* **sp. nov.** differs from other stout‐bodied species (*G. orientalis* **sp. nov.** and *G. willdenowi*) by a posterior opercular edge with pointed upper tip and rounded lower edge (*vs*. posterior opercular edge w‐shaped with two equally long tips) and a reduced pigmentation. In addition, it differs from *G. orientalis* **sp. nov.** by principal caudal‐fin rays 12–13 (*vs*. principal caudal rays 10–11) and from *G. willdenowi* by vertical eye diameter 3.4–4.3% and horizontal eye diameter 3.0–3.7% of standard length (*vs*. vertical eye diameter 2.6–3.3% and horizontal eye diameter 2.3–2.9% of standard length). *G. adriatica* **sp. nov.** is known from the Adriatic Sea as well as Corfu island and has no overlapping geographic records with *G. orientalis* **sp. nov.** and *G. willdenowi*.

#### 3.1.3 *Gouania orientalis* sp. nov.

**English name:** **Oriental** **blunt‐snouted** **clingfish**

*ZooBank LSID*: urn:lsid:zoobank.org:act:4E02C972‐6907‐41E0‐A2B7‐0215FDB49319

**FIGURE 6:**
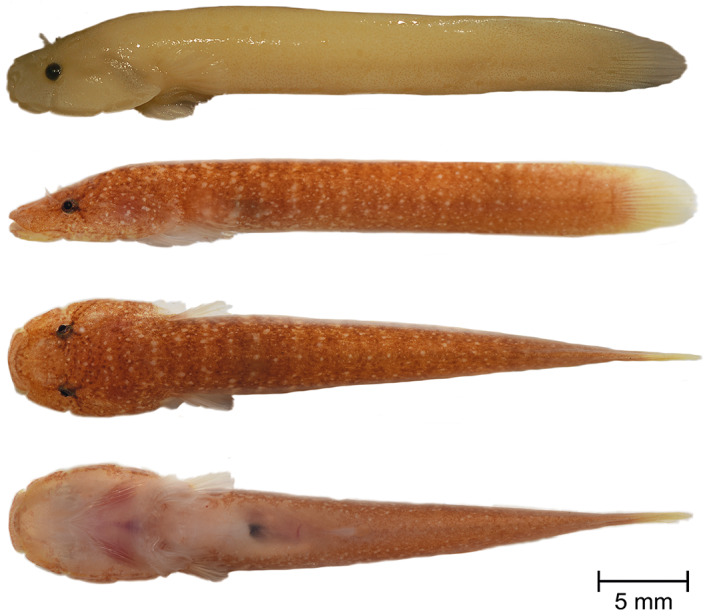
*Gouania orientalis* **sp. nov.**, PMR VP4585, holotype, male, 32.8+4.93 mm, Plakias, Crete, Greece. Lateral view of specimen preserved in 4% formaldehyde (top). Lateral, dorsal and ventral view, alive (below). Photographs by M. Wagner and M. Kovačić

**Holotype.** PMR VP4585, male, 32.8 + 4.93 mm, Plakias, Crete, Greece, 35°11′40.8″N, 24°22′50.9″E, coll. M. Wagner, August 9, 2016 (Figure 6).

**Paratypes.** PMR VP4719, male, 37.53 + 5.59 mm, Gulf of Corinth, Greece, 38°10′17.0″N, 22°16′26.7″E, coll. M. Wagner, August 23, 2018; PMR VP4584, female, 26.0 + 4.56 mm, Plakias, Crete, Greece, 35°11′40.8″N, 24°22′50.9″E, coll. M. Wagner, August 9, 2016; ZSM‐PIS‐047658, female, 30.55 + 5.25 mm and PMR VP4588, juvenile of unidentified sex, 17.92 + 2.98 mm, both from Vatos, Crete, Greece, 34°59′41.0″N, 25°33′17.3″E, coll. M. Wagner, August 13, 2016; ZSM‐PIS‐047659, female, 17.88 + 2.79 mm and PMR VP4596, female, 17.09 + 2.69 mm, both from Souda Beach, Plakias, Crete, Greece, 35°11′32.1″N, 24°22′04.9″E, coll. M. Wagner, August 17, 2016; ZSM‐PIS‐047660, female, 24.87 + 3.56 mm and ZSM‐PIS‐047660, female, 23.68 + 3.39, mm, both from Chamolia, Greece, 37°54′58.5″N, 24°02′08.7″E, coll. M. Wagner, August 16, 2018; ZSM‐PIS‐047661, male, 28.52 + 4.04 mm, Feloti Beach, Kapsáli, Kythira, Greece, 36°09′16.2″N, 22°57′52.0″E, coll. M. Wagner, August 21, 2018.

**Diagnosis.** *Gouania orientalis* **sp. nov.** differs from its congeners by the combination of the following characters: (1) dorsal head profile a straight line from nape above eye to upper lip tip; (2) posterior angle of jaws extends to between a vertical line drawn through anterior edge of eye and a vertical line drawn through anterior part of eye; (3) infraorbital invagination vertical to posterior part of eye; (4) posterior opercular edge w‐shaped with two equally long tips; (5) longitudinal infralateral and suborbital transversal rows of superficial neuromasts placed in the well‐defined deep groove; (6) trunk cross‐section behind pectoral fin base half oval with straight ventral side; (7) granules on body shallow and inconspicuous; (8) upper attachment of gill membrane opposite to 5th to 6th pectoral ray; (9) pectoral rays 17–19; (10) upper attachment of disc membrane attaching to base of pectoral fin at 16th–18th pectoral ray; (11) principal caudal rays 10–11; (12) head length 25.0–28.9% of standard length; (13) pectoral fin length 8.2–10.3% of standard length; (14) prepectoral distance 24.7–28.2% of standard length; (15) ventral adhesive disc length 14.9–19.0% of standard length; (16) caudal‐fin length 14.2–17.5% of standard length; (17) low number of vertebrae (Supporting Information Table S2; 35–36) (18) pharyngeal jaws with small ceratobranchial 5, having a few hardly recognizable small conical teeth; (19) nasal bones with inconspicuous frontal end; (20) in life star‐like pigmentation around eyes, body colouration dark, sometimes marbled or with stripes.

**Description.** *General morphology*: Body proportions are given in Table 1. Body slender and elongated, posteriorly laterally compressed, body depth at pectoral fins 6.9–8.6 in SL, body depth at anus 7.8–9.3 in SL, body depth in width at pectoral fins 1.1–1.2, body depth in width at anus 0.8–0.9. Trunk cross‐section behind pectoral fin base half oval with straight ventral side. Granules on body shallow and inconspicuous. Head dorsoventrally compressed, head depth in width at orbit 1.5–1.9 and moderately large, head length 3.5–4.0 in SL, head wider than body width maximum, head width at anterior sucking disc edge 0.7–0.8 in body width at pectoral fins. Dorsal head profile straight between nape above eye and upper lip tip. Head rounded in dorsal view. Snout large compared to eyes, preorbital distance 3.1–3.8 in head length, 0.3–0.5 in horizontal eye diameter. Snout wide, not produced, blunt. Internostril space gently convex. Eyes dorsolateral, with lower eye edge rounded. Eyes small, 7.0–10.1 in head length, vertical diameter of the eye 0.8–1.1 in horizontal eye diameter. Infraorbital invagination vertical to posterior part of eye. Interorbital distance wide, 0.3–0.5 in horizontal eye diameter. Centre of eye much closer to tip of snout than to posterior margin of operculum, preorbital distance in postorbital distance 1.9–2.3. Anterior and posterior nostrils long tubes of about equal length. Nostrils well separated and posterior nostril located behind and dorsally to anterior edge of eyes. Single large dermal flap at the posterior margin of anterior nostril leaf shaped, longer than nostril. Posterior nostril rim slightly crenate with no extension. Head lateral line system with canals with pores and with superficial neuromasts arranged in rows. Head canals reduced and pores small. Single pore in nasal canal near posterior nostril. Single pore in postorbital canal close to posterior eye edge. Two pores in mandibular canal, anterior one close to anteriolateral angle of mouth, posterior pore slightly in front of vertical of posterior angle of jaws, posterior pore usually more prominent. Lachrymal as well as preopercular canals and pores absent. Rows of superficial neuromasts as follows: SR 2, NR 3–5, LIR 23–28, STR 3–4, POR 3–4, PTR 2, SLR 5, MR 9–13, AVR 2, PVR 1, ADR 1–3, PDR 1, HR 3, SR1 3, SR2 1–2, DLR 6–8, VLR 9–13. STR and LIR rows of superficial neuromasts placed in the well‐defined deep groove. DLR row of superficial neuromasts anteriorly starts above pectoral fin, continuously dorsolateral and ends posteriorly downwards at midlateral level and variably, vertical to anus or in front of it or behind it. VLR anteriorly starts behind pectoral fin base, continuous ventrolaterally and ends posteriorly upwards with last papilla nearly at midlateral level at caudal fin base or close to it. Mouth terminal, upper and lower lips end about equally, lips fleshy, upper lip larger than the lower lip. Posterior angle of jaws extends to between vertical line drawn through anterior edge of eyes and vertical line drawn through anterior part of eye. Chin with bilobed or slightly bilobed fold at anterior edge covering MR row of superficial neuromasts. The gill membrane is attached to isthmus, gill opening starting at the base of pectoral fin, with the upper attachment of the gill membrane is opposite to 5th to 6th pectoral ray. Posterior opercular edge w‐shaped with two equally long tips. No subopercular spine. No fleshy pad present on lower pectoral base. Urogenital papilla present. Preanus length in postanus length 0.6–0.8. Anal papillae absent, the area around anus wrinkled.

*Fins*. Rudimentary dorsal and anal fins located well posteriorly and short, reduced to low ridges with very weak rays, connected to the caudal fin. Pectoral rays 17–19. Caudal fin rounded, principal caudal rays 10–11. Ventral adhesive disc (Figure 4b) of “double” type, anterior margin crenate with large invagination on each lateral side and central invagination at midventral; posterior margin slightly crenate. Disc small, disc length 5.3–6.7 in SL, its width slightly larger than its length, width in length 0.9–1.1. No papillae in region A and flattened papillae in regions B and C. In region B two to three rows of papillae with total papillae count 21–37 and in region C two rows of papillae with total papillae count 9–15. No inner row of papillae on lateral sides of the central part of the anterior disc. Upper attachment of disc membrane attaching to base of pectoral fin at 16th–18th pectoral ray (*i.e*., on penultimate ray).

*Colouration*. Background colour of live specimens bright yellow to brownish red (Figure 6) and prominent star‐like pigmentation around eyes present. Body pigments reduced (juveniles) or, behind head, with clearly visible regular stripes (juveniles, *e.g*., Attica, Crete, Kythira) or marbled (*i.e*., irregular pattern; *e.g*., Gulf of Corinth). Specimens from Crete, Kythira and the Gulf of Corinth have stronger pigmentation (see Figure 6), hence, the pigmentation pattern can be less clearly visible. Formaldehyde fixed specimens white‐yellow and without pigments. Ethanol fixed specimens white to skin‐coloured, pigmentation present (also stripes). For more pictures of life colouration see Supporting Information File S1.

*Dentition and osteology*. Upper jaw with outer row of about 10 (one side) medium‐sized caniniforms frontally. Behind them inner small conical teeth irregularly scattered in two separate (left and right) drop‐like patches medially wide about 5–6 teeth, becoming narrowed to a single row of teeth laterally. Outer row continues laterally as two large caniniforms, followed behind by about eight medium‐sized caniniforms. Lower jaw with outer row of about 10 (one side) medium‐sized caniniforms frontally. Behind them single broad patch of small conical inner teeth wide medially about 5–6 teeth, becoming narrowed to a single row of teeth laterally. The single row of about six larger caniniforms continuous laterally. Pharyngeal jaws with small ceratobranchial 5, having a few (1–2) poorly recognizable small conical teeth (Figure 5b), pharyngobranchial 3 toothplate not visible on 3D models from microCT images. Number of vertebrae 35–36, abdominal 16 and caudal 21 (Supporting Information Table S2). The first gill arch with hemibranch, the 2nd to 4th gill arches with holobranchs. Subopercle indistinguishable from opercle, shaped as its posterior elongated extension, not forming or having subopercular spine. Six branchiostegals. Nasal bones with inconspicuous frontal end. Maxillary, premaxillary, nasal and ceratobranchial 5 bones shaped as on Figure 5b.

**Etymology.** Named *orientalis*, from the Latin word “oriens” for “east”, which describes the distribution range of the species that is restricted to the oriental Mediterranean basin. The name is an adjective in the nominative singular (Article 11.9.1.1., ICZN, 1999).

**Ecology and geographical distribution (Figure** **1** **a).** The known species distribution range encompasses the Gulf of Corinth, the Aegean Sea (Attica) and on the islands Crete and Kythira. Quantitative data on ecology is largely lacking*. Gouania orientalis* **sp. nov.** occurs in sympatry or even syntopy with *G. hofrichteri* **sp. nov.** throughout its distribution range. The species inhabits intertidal and subtidal pebble and boulder beaches.

**Remarks.** *Gouania orientalis* **sp. nov.** differs from slender‐bodied *Gouania* species (*G. pigra* and *G. hofrichteri* **sp. nov.**) by a dorsal head profile that forms a straight line between nape above eye and upper lip tip (*vs*. dorsal head profile in lateral view “S” curved, concave above eye and convex at nape), an infraorbital invagination vertical to posterior part of eye (*vs*. infraorbital invagination below anterior half of eye or below mideye), lower number of vertebrae (Supporting Information Table S2; 35–36 *vs*. 38–40) and a star‐like pigmentation around eyes (*vs*. no star‐like pigmentation around eyes). Nine morphometric characters as percentages of standard length of *G. orientalis* **sp. nov.** are nonoverlapping in range with both slender‐bodied *Gouania*: head length, horizontal eye diameter, body width at pectoral fins, pectoral‐fin length, prepectoral distance, ventral adhesive disc length, distance between the posterior margin of sucking disc and anus, caudal base depth and caudal‐fin length (values in Table 1). There are also morphometric characters nonoverlapping in range with only one of the two slender‐bodied *Gouania* (Table 1). In addition, *G. orientalis* **sp. nov.** differs from *G. pigra* by a posterior angle of jaws extending to between vertical line drawn through anterior edge of eye and vertical line drawn through anterior part of eye (*vs*. posterior angle of jaws extending to, or close to, a vertical line drawn through the anterior edge of the anterior nostril), pectoral rays 17–19 (*vs*. pectoral rays 13–16) and upper attachment of disc membrane attaching to base of pectoral fin at 16th–18th pectoral ray (*vs*. upper attachment of disc membrane attaching to base of pectoral fin at 12th–15th pectoral ray). *G. orientalis* **sp. nov.** is also different from *G. hofrichteri* **sp. nov.** by posterior opercular edge w‐shaped with two equally long tips (*vs*. pointed upper tip and rounded lower posterior opercular edge), longitudinal infralateral and suborbital transversal rows of superficial neuromasts placed in the well‐defined deep groove (*vs*. longitudinal infralateral and suborbital transversal rows of superficial neuromasts placed in shallow groove disappearing in posterior part of longitudinal infralateral row), body cross‐section behind pectoral fin base half oval with straight ventral side (*vs*. trunk cross‐section behind pectoral fin base triangular with ventral flat and dorsal pointed), granules on body shallow and inconspicuous (*vs*. granules on body, at least on posterior part and nape, large and prominent) and upper attachment of gill membrane opposite to 5th–6th pectoral ray (*vs*. the upper attachment of the gill membrane opposite to 3rd–4th pectoral ray). *G. orientalis* **sp. nov.** differs from the stout‐bodied species *G. adriatica* **sp. nov.** in posterior opercular edge w‐shaped with two equally long tips (*vs*. posterior opercular edge with pointed upper tip and rounded lower edge), principal caudal rays 10–11 (*vs*. principal caudal‐fin rays 12–13) and pattern of pigmentation (stripes, marbles *vs*. reduced pigmentation). *G. orientalis* **sp. nov.** has no nonoverlapping external morphological differences to *G. willdenowi* but differs by its low number of vertebrae (Supporting Information Table S2; 35–36 *vs*. 37–38). *G. orientalis* **sp. nov.** is known from Aegean and Ionian Sea and has nonoverlapping geographic distribution with *G. adriatica* **sp. nov.**, *G. pigra* and *G. willdenowi*.

#### 3.1.4 *Gouania hofrichteri* sp. nov.

**English name: Hofrichter's clingfish**

*ZooBank LSID*: urn:lsid:zoobank.org:act:3894FA6B‐E67C‐4905‐A31C‐F6FD1EF4F2A8

**FIGURE 7:**
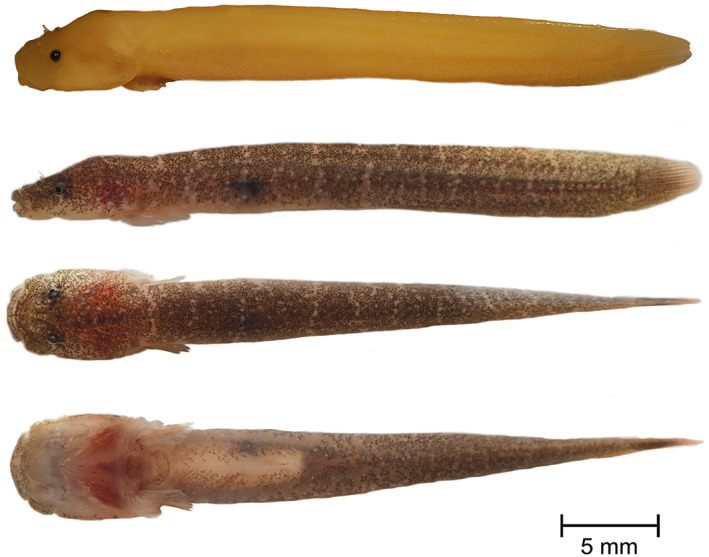
*Gouania hofrichteri* **sp. nov.**, PMR VP4595, holotype, male, 30.35+3.67 mm, Souda Beach, Plakias, Crete, Greece. Lateral view of specimen preserved in 4% formaldehyde (top). Lateral, dorsal and ventral view, alive (below). Photographs by M. Wagner and M. Kovačić

**Holotype.** PMR VP4595, male, 30.35 + 3.67 mm, Souda Beach, Plakias, Crete, Greece, 35°11′32.1″N, 24°22′04.9″E, coll. M. Wagner, August 17, 2016 (Figure 7).

**Paratypes**. PMR VP4591, female, 21.42 + 2.62 mm, Mades, Crete, Greece, 35°24′01.1″N, 25°02′01.6″E, coll. M. Wagner, August 15, 2016; PMR VP4599, female, 26.8 + 3.23 mm and PMR VP4600, male, 29.75 + 3.49 mm, both from Souda Beach, Plakias, Crete, Greece, 35°11′32.1″N, 24°22′04.9″E, coll. M. Wagner, August 17, 2016; PMR VP4605, juvenile of unidentified sex, 20.62 + 2.63 mm, Saronida, Greece, 37°43′12.2″N, 23°55′41.0″E, coll. M. Wagner, August 2, 2016; PMR VP4606, female, 29.47 + 3.41 mm, ZSM‐PIS‐047656, female, 35.86 + 4.49 mm and PMR VP4608, female, 26.69 + 3.32 mm, all from Chamolia, Greece, 37°54′58.5″N, 24°02′08.7″E, coll. M. Wagner, August 4, 2016; ZSM‐PIS‐047657, male, 36.91 + 4.24 mm and ZSM‐PIS‐047657, male, 33.27 + 4.31, mm, both from Chamolia, Greece, 37°54′58.5″N, 24°02′08.7″E, coll. M. Wagner, August 16, 2018.

**Diagnosis.** *Gouania hofrichteri* **sp. nov.** differs from congeneric species by the combination of the following characters: (1) dorsal head profile in lateral view “S” curved, concave above eye and convex at nape; (2) posterior angle of jaws extends to between vertical line drawn through posterior edge of anterior nostril and vertical line drawn through anterior edge of eye; (3) infraorbital invagination below anterior half of eye or below mideye; (4) posterior opercular edge with pointed upper tip and rounded lower edge; (5) longitudinal infralateral and suborbital transversal rows of superficial neuromasts placed in shallow groove disappearing in posterior part of longitudinal infralateral row; (6) body cross‐section behind pectoral fin base triangular with ventral flat and dorsal pointed; (7) granules on body, at least on posterior part and nape, large and prominent; (8) upper attachment of gill membrane opposite to 3rd–4th pectoral ray; (9) vertical eye diameter 2.0–2.7% of standard length; (10) head length 18.9–23.4% of standard length; (11) pectoral‐fin length 5.4–6.4% of standard length; (12) prepectoral distance 19.5–23.7% of standard length; (13) ventral adhesive disc length 10.2–13.4% of standard length; (14) caudal‐fin length 11.5–13.4% of standard length; (15) high total number of vertebrae (Supporting Information Table S2; 38–40); (16) pharyngeal jaws with ceratobranchial 5 elongated, having several larger elongated conical teeth; (17) nasal bones hook‐shaped; (18) star‐like pigmentation around eyes absent, body pigmentation striped or marbled, small iridophores visible.

**Description.** *General morphology*: Body proportions are given in Table 1. Body very slender and elongated, posteriorly laterally compressed, body depth at pectoral fins 9.2–10.5 in SL, body depth at anus 11.0–13.5 in SL, body depth in width at pectoral fins 1.0–1.0, body depth in width at anus 0.7–0.9. Body cross‐section behind pectoral fin base triangular with ventral flat and dorsal pointed. Granules on body, at least on posterior part and nape, large and prominent. Head dorsoventrally compressed, head depth in width at orbit 1.5–1.8, and moderately small, head length 4.3–5.3 in SL, head wider than body width maximum, head width at anterior sucking disc edge 0.7–0.8 in body width at pectoral fins. Dorsal head profile “S” curved, concave above eye and convex at nape. Head rounded in dorsal view. Snout large compared to eyes, preorbital distance 2.8–3.7 in head length, 0.3–0.5 in horizontal eye diameter. Snout wide, not produced, blunt. Internostril space almost triangular in cross‐section, with ridge top, conspicuously convex. Eyes dorsolateral, with lower eye edge rounded. Eyes small, 7.7–10.7 in head length, vertical diameter of the eye 0.8–1.1 in horizontal eye diameter. Infraorbital invagination below anterior half of eye or below mideye. Interorbital distance wide, 0.3–0.5 in horizontal eye diameter. Centre of eye much closer to tip of snout than to posterior margin of operculum, preorbital distance in postorbital distance 1.7–2.3. Anterior and posterior nostrils long tubes of about equal length. Nostrils well separated and posterior nostril located behind and dorsally to the anterior edge of eyes. Single large dermal flap at the posterior margin of anterior nostril leaf shaped, longer than nostril. Posterior nostril rim crenate with no extension. Head lateral line system with canals with pores and with superficial neuromasts arranged in rows. Head canals reduced and pores small. Single pore in nasal canal near posterior nostril. Single pore in postorbital canal close to posterior eye edge. Two pores in mandibular canal, anterior one close to anteriolateral angle of mouth, posterior pore slightly in front of vertical of posterior angle of jaws, posterior pore usually more prominent. Lachrymal as well as preopercular canals and pores absent. Rows of superficial neuromasts as follows: SR 2, NR 3, LIR 22–29, STR 2–4, POR 3, PTR 2, SLR 4–5, MR 9–11, AVR 2, PVR 1, ADR 2–3, PDR 1, HR 3–4, SR1 3–4, SR2 1–2, DLR 5–8, VLR 10–13. STR and LIR rows of superficial neuromasts placed in shallow groove disappearing in posterior part of LIR. DLR row of superficial neuromasts anteriorly starts above pectoral fin, continuously dorsolateral and ends posteriorly downwards at or above midlateral level vertical to anus. VLR anteriorly starts behind pectoral fin base, continuous ventrolaterally and ends posteriorly upwards with last papilla near or at midlateral level at or close to caudal‐fin base. Mouth terminal, upper and lower lips ends about equally, lips fleshy, upper lip larger than the lower lip. Posterior angle of jaws extends to between vertical line drawn through posterior edge of anterior nostril and vertical line drawn through anterior edge of eye. Chin with bilobed or single lobe fold at anterior edge not covering MR row of superficial neuromasts. The gill membrane is attached to isthmus, gill opening starting at the base of pectoral fin, with the upper attachment of the gill membrane is opposite to 3rd–4th pectoral ray. Posterior opercular edge with pointed upper tip and rounded lower edge. No subopercular spine. No fleshy pad present on lower pectoral base. Urogenital papilla present. Preanus length in postanus length 0.7–1.0. Anal papillae absent, the area around anus wrinkled.

*Fins*. Rudimentary dorsal and anal fins located well posteriorly and short, reduced to low ridges with the very weak rays, connected to the caudal fin. Pectoral rays 14–16. Caudal fin rounded, principal caudal rays 10–12. Ventral adhesive disc (Figure 4c) of “double” type, anterior margin crenate with large invagination on each lateral side and central invagination at midventral; posterior margin crenate. Disc very small, disc length 7.5–9.8 in SL, its width slightly larger than its length, width in length 0.8–0.9. No papillae in region A and flattened papillae in regions B and C. In region B one or two rows of papillae with total papillae count 12–30 and in region C one or two rows of papillae with total papillae count 4–14. No inner row of papillae on lateral sides of the central part of the anterior disc. Upper attachment of disc membrane attaching to base of pectoral fin at 14th–16th pectoral ray (*i.e*., at ultimate or penultimate ray). Males can have two prominent seemingly perfused finger‐like extensions on each site of sucking disc that are of equal size or exceeding length of disc region A (Figure 4f).

*Colouration*. Background colour of live specimens bright to skin‐coloured (Figure 7). Pigmentation behind head region sometimes with a clearly visible striped or marbled (*i.e*., irregularly distributed patches of pigments) pattern. Sometimes small evenly distributed iridophores visible in lateral view (Figure 7) in life. No star‐like pigmentation around the eyes visible. Formaldehyde fixed specimens white‐yellow and without pigments. Ethanol fixed specimens white to skin coloured, striped or marbled pigmentation visible. For more pictures of live colouration see Supporting Information File S1.

*Dentition and osteology*. Upper jaw with outer row of about eight (one side) medium‐sized caniniforms frontally. Behind them inner small conical teeth irregularly scattered in two separate (left and right) drop‐like patches medially wide about 5–6 teeth, becoming narrowed to a single row of teeth laterally. Outer row continues laterally laterally as four large caniniforms of variable size, followed behind by about six medium‐sized caniniforms. Lower jaw with outer row of eight to 10 (one side) medium‐sized caniniforms frontally. Behind them single broad patch of small conical inner teeth medially wide about 5–6 teeth, becoming narrowed to a single row of teeth laterally. The single row of about five larger caniniforms continuous laterally. Pharyngeal jaws with elongated ceratobranchial 5, having several (about 5) larger elongated conical teeth (Figure 5c), pharyngobranchial 3 toothplate not visible on 3D models from micro‐computed tomography (microCT) images. Number of vertebrae 38–40, abdominal 17 and caudal 21–22 (Supporting Information Table S2). The first gill arch with hemibranch, the 2nd to 4th gill arches with holobranchs. Subopercle indistinguishable from opercle, shaped as its posterior elongated extension, not forming or having subopercular spine. Six branchiostegals. Hook‐shaped nasal bones. Maxillary and premaxillary bones shaped as on Figure 5c.

**Etymology.** Named *hofrichteri*, in honour of Robert Hofrichter, whose work on European clingfishes sparked our interest in these enigmatic fishes. The species epithet was formed from the personal name, as the noun in the genitive case, with “i” added to the stem of the name (Article 31.1.2., ICZN, 1999).

**Ecology and geographical distribution (Figure** **1** **a).** Species widespread in the eastern Mediterranean Sea with a single record from the Adriatic Sea (Pelješac; Figure 1c). *G. hofrichteri* **sp. nov**. is very abundant in the northern and southern Ionian Sea (Corfu, Stomio), the Gulf of Corinth, the islands of Kythira and Crete and the Aegean Sea (Attica). Quantitative data on ecology is largely lacking. The species inhabits intertidal pebble beaches and probably shows passive emergence behaviour (compare with Bilecenoğlu, 2015). Throughout its distribution range this species occurs in sympatry with *G. orientalis* **sp. nov.** and on the island Corfu with *G. adriatica* **sp. nov**. There is a single record of the species inside the Adriatic basin from Pelješac, where it occurs in low densities together with *G. pigra* and *G. adriatica* **sp. nov**.

**Remarks.** *Gouania hofrichteri* **sp. nov.** differs from all other *Gouania* species by longitudinal infralateral and suborbital transversal rows of superficial neuromasts placed in shallow groove disappearing in posterior part of longitudinal infralateral row (*vs*. longitudinal infralateral and suborbital transversal rows of superficial neuromasts placed in the well‐defined deep groove in all other *Gouania* species); body cross‐section behind pectoral fin base triangular with ventral flat and dorsal pointed (*vs*. body cross‐section behind pectoral fin base half oval to pentagonal (pentagonal only in *G. pigra*) with straight ventral side in all other *Gouania* species); granules on body, at least on posterior part and nape, large and prominent (*vs*. the granules on body shallow and inconspicuous in all other *Gouania* species) and hook‐like nasal bones. *G. hofrichteri* **sp. nov.** differs from three stout‐bodied *Gouania* species (*G. adriatica* **sp. nov.**, *G. orientalis* **sp. nov.** and *G. willdenowi*) by dorsal head profile in lateral view “S” curved, concave above eye and convex at nape (*vs*. dorsal head profile straight between nape above eye and upper lip tip), absence of star‐like pigmentation around eyes and large number of vertebrae (Supporting Information Table S2; 38–40 *vs*. 35–38). Eleven morphometric characters as percentages of standard length of *G. hofrichteri* **sp. nov.** are nonoverlapping in range with all three stout‐bodied *Gouania*: vertical eye diameter, head length, postorbital distance, all three head widths, body width at pectoral fins, pectoral‐fin length, prepectoral distance, ventral adhesive disc length and caudal‐fin length (values in the Table 1). In addition, there are numerous morphometric characters nonoverlapping in range with one or two out of three stout‐bodied *Gouania* species (Table 1). *G. hofrichteri* **sp. nov.** differs from *G. adriatica* **sp. nov.** and *G. orientalis* **sp. nov.** by the upper attachment of the gill membrane opposite to 3rd–4th pectoral ray (*vs*. upper attachment of the gill membrane opposite to 5th–6th pectoral ray) and from *G. orientalis* **sp. nov.** and *G. willdenowi* by the infraorbital invagination below anterior half of eye or below mideye (*vs*. infraorbital invagination vertical to posterior part of eye) and by posterior opercular edge with pointed upper tip and rounded lower edge (*vs*. posterior opercular edge w‐shaped with two equally long tips). *G. hofrichteri* **sp. nov.** differs from other slender‐bodied species, *G. pigra*, by the posterior angle of jaws extending to between vertical line drawn through posterior edge of anterior nostril and vertical line drawn through anterior edge of eye (*vs*. posterior angle of jaws extending to, or close to, vertical line drawn through anterior edge of anterior nostril) and by posterior opercular edge with pointed upper tip and rounded lower edge (*vs*. posterior opercular edge with two tips, upper longer or equal to lower). *G. hofrichteri* **sp. nov.** is known from the Adriatic (single findings from Pelješac), Aegean and Ionian Sea and has nonoverlapping geographic distribution only with *G. willdenowi*.

#### 3.1.5 *Gouania pigra* (Nardo 1827)

**English name: Piglet sucker**

**FIGURE 8:**
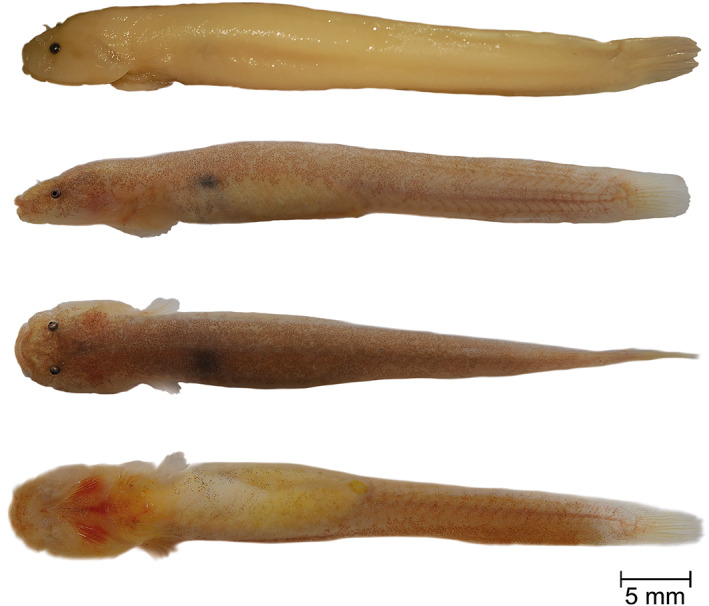
*Gouania pigra* (Nardo, 1827), PMR VP3529, neotype, female, 43.12+5.03 mm, Glavotok, Krk, Croatia. Lateral view of specimen preserved in 4% formaldehyde (top). Lateral, dorsal and ventral view, alive (below). Photographs by M. Wagner and M. Kovačić

**Neotype.** PMR VP3529, female, 43.12 + 5.03 mm, Glavotok, Krk, Croatia, 45°05′44.9″N, 14°26′32.4″E, coll. M. Wagner, May 16, 2015 (Figure 8).

**Additional material examined.** ZSM‐PIS‐047648, male, 37.71 + 4.36 mm, ZSM‐PIS‐047648, male, 37.2 + 4.31 mm and ZSM‐PIS‐047648, female, 32.58 + 3.94 mm, all from Stara Baška, Krk, Croatia, 44°56′45.3″N, 14°42′22.2″E, coll. M. Wagner, September 16, 2019; PMR VP3531, male, 39.48 + 4.4 mm, Glavotok, Krk. Croatia, 45°05′44.9″N, 14°26′32.4″E, coll. M. Wagner, May 16, 2015; PMR VP4619, female, 35.86 + 4.14 mm, Stoja, Pula, Croatia, 44°51′38.4″N, 13°49′05.0″E, coll. M. Wagner, July 17, 2016; ZSM‐PIS‐047649, female, 35.14 + 4.03 mm and ZSM‐PIS‐047649, male, 37.07 + 4.51 mm, both from Pećine, Rijeka, Croatia, 45°18′52.4″N, 14°28′11.7″E, coll. M. Wagner, July 12, 2019; PMR VP4581, male, 38.67 + 4.91 mm and PMR VP4583, male, 36.87 + 4.08 mm, Sv. Marina, Istria, Croatia, 45°01′42.1″N, 14°09′17.4″E, coll. M. Wagner, July 18, 2015.

**Synonyms.** *Lepadogaster piger* Nardo 1827: 9 (original description; type locality: Rovinj; holotype: unknown); *Gouania prototypus* Nardo 1833: 548 (original description; type locality: Rovinj?; holotype: unknown), *Gouania piger* Bonaparte 1846: 64 (original description; type locality: unknown; holotype: unknown); *Leptopterygius piger* Günther 1861: 515 (original description; type locality: unknown; holotype: unknown).

**Diagnosis.** *Gouania pigra* differs from the congeneric species by the combination of the following characters: (1) dorsal head profile in lateral view “S” curved, concave above eye and convex at nape; (2) posterior angle of jaws extends to, or close to, vertical line drawn through anterior edge of anterior nostril; (3) infraorbital invagination below anterior half of eye or below mideye; (4) posterior opercular edge with two tips, upper longer or equal to lower; (5) longitudinal infralateral and suborbital transversal rows of superficial neuromasts placed in the well‐defined deep groove; (6) body cross‐section behind pectoral fin base half oval to pentagonal with straight ventral side; (7) granules on body shallow and inconspicuous; (8) pectoral rays 13–16; (9) upper attachment of disc membrane attaching to base of pectoral fin at 12th–15th pectoral ray; (10) principal caudal rays 10–11; (11) head length 19.5–22.9% of standard length; (12) pectoral‐fin length 5.6–7.5% of standard length; (13) prepectoral distance 19.5–23.4% of standard length; (14) ventral adhesive disc length, 12.1–14.6% of standard length; (15) caudal‐fin length 11.1–12.7% of standard length; (16) large total number of vertebrae (Supporting Information Table S2; 39–40); (17) pharyngeal jaws with elongated ceratobranchial 5, having several larger elongated conical teeth; (18) nasal bones hook‐shaped; (19) no star‐like pigmentation around eyes, pigmentation generally reduced.

**Description.** *General morphology*: Body proportions are given in Table 1. Body very slender and elongated, posteriorly laterally compressed, body depth at pectoral fins 8.5–9.4 in SL, body depth at anus 8.8–11.8 in SL, body depth in width at pectoral fins 1.0–1.2, body depth in width at anus 0.7–0.9. Body cross‐section behind pectoral fin base half oval to pentagonal with straight ventral side. Granules on body shallow and inconspicuous. Head dorsoventrally compressed, head depth in width at orbit 1.5–1.9, and moderately small, head length 4.4–5.1 in SL, head wider than body width maximum, head width at anterior sucking disc edge 0.7–0.8 in body width at pectoral fins. Dorsal head profile “S” curved, concave above eye and convex at nape. Head rounded in dorsal view. Snout large compared to eyes, preorbital distance 2.7–3.6 in head length, 0.3–0.4 in horizontal eye diameter. Snout wide, not produced, blunt. Internostril space convex to gently convex. Eyes dorsolateral, rounded or drop‐like with slightly pointed lower eye edge. Eyes small, 9.1–11.2 in head length, vertical diameter of the eye 0.7–0.9 in horizontal eye diameter. Infraorbital invagination below anterior half of eye or below mideye. Interorbital distance wide, 0.3–0.4 in horizontal eye diameter. Centre of eye much closer to tip of snout than to posterior margin of operculum, preorbital distance in postorbital distance 1.8–2.4. Anterior and posterior nostrils long tubes of about equal length. Nostrils well separated and posterior nostril located behind and dorsally to the anterior edge of eyes. Single large dermal flap of leaf shape at the posterior margin of anterior nostril, longer than nostril. Posterior nostril rim crenate or villose with no extension. Head lateral line system with canals with pores and with superficial neuromasts arranged in rows. Head canals reduced and pores small. Single pore in nasal canal near posterior nostril. Single pore in postorbital canal close to posterior eye edge. Two pores in mandibular canal, anterior one close to anteriolateral angle of mouth, posterior pore slightly in front of vertical of posterior angle of jaws, posterior pore usually more prominent. Lachrymal as well as preopercular canals and pores absent. Rows of superficial neuromasts as follows: SR 2, NR 3, LIR 21–26, STR 2–3, POR 2–3, PTR 2, SLR 5, MR 8–12, AVR 2, PVR 1, ADR 3, PDR 1–2, HR 3, SR1 3, SR2 1–2, DLR 6–10, VLR 12–16. STR and LIR rows of superficial neuromasts placed in the well‐defined deep groove. DLR row of superficial neuromasts anteriorly starts above pectoral fin, continuously dorsolateral and ends posteriorly variably: downwards at midlateral level or above it, at vertical from anus, or in front or behind it. VLR anteriorly starts behind pectoral fin base, continuously ventrolateral and ends posteriorly upwards with last papilla nearly at midlateral level at caudal fin base or more distant from it. Mouth terminal, upper and lower lips end about equally, lips fleshy, upper lip larger than the lower lip. Posterior angle of jaws extends to, or close to, a vertical line drawn through the anterior edge of the anterior nostril. Chin with bilobed or single lobed fold at anterior edge covering MR row of superficial neuromasts. Gill membrane is attached to isthmus, gill opening starting at base of pectoral fin, with upper attachment of gill membrane opposite to 3rd to 5th pectoral ray. Posterior opercular edge with two tips, upper longer or equal to lower. No subopercular spine. No fleshy pad present on lower pectoral base. Urogenital papilla present. Preanus length in postanus length 0.7–0.9. Anal papillae absent, the area around anus wrinkled.

*Fins*. Rudimentary dorsal and anal fins located well posteriorly and short, reduced to low ridges with very weak rays, connected to caudal fin. Pectoral rays 13–16. Caudal fin rounded, principal caudal rays 10–11. Ventral adhesive disc (Figure 4d) of “double” type, anterior margin crenate or straight with large invagination on each lateral side and central invagination at midventral; posterior margin crenate or straight. Disc very small, disc length 6.9–8.3 in SL, its width slightly larger than its length, width in length 0.8–1.0. No papillae in region A and flattened papillae in regions B and C. In region B one or two rows of papillae with total papillae count 11–23 and in region C one or two rows of papillae with total papillae count 4–11. No inner row of papillae on lateral sides of the central part of the anterior disc. Upper attachment of disc membrane attaching to base of pectoral fin at 12th–15th pectoral ray, *i.e*., at ultimate or penultimate ray. Males can have two prominent seemingly perfused finger‐like extensions on each site of sucking disc that sometimes equal or exceed length of disc region A (Figure 4f).

*Colouration*. Background colouration of live specimens white to flesh‐coloured, slightly transparent (Figures 1b and 8) and no star‐shaped pigmentation around eyes. In life body almost pigmentless or with very small pigments, leading to an irregular marbled pattern, but never as strong as in other *Gouania* species (Supporting Information File S1). Formaldehyde fixed specimens white‐yellow and without pigments. Ethanol fixed specimens white to skin‐coloured pigmentation reduced. For more pictures of life colouration see Supporting Information File S1.

*Dentition and osteology*. Upper jaw with outer row of about eight (one side) medium‐sized caniniforms frontally. Behind them inner small conical teeth irregularly scattered in two separate (left and right) drop‐like patches medially wide about 5–6 teeth, becoming narrowed to a single row of teeth laterally. Outer row continues laterally as four large caniniforms of variable size, followed behind by four to five medium‐sized caniniforms. Lower jaw with outer row of 10 to 12 (one side) medium‐sized caniniforms frontally. Behind them single broad patch of small conical inner teeth medially wide about 5–6 teeth, becoming narrowed to a single row of teeth laterally. The single row of about six larger caniniforms continuous laterally. Pharyngeal jaws with elongated ceratobranchial 5, having several (about 4–5) larger elongated conical teeth (Figure 5d), pharyngobranchial 3 toothplate not visible on 3D models from microcomputed tomography (microCT) images. Number of vertebrae 39–40, abdominal 17 and caudal 22–23 (Supporting Information Table S2). The first gill arch with hemibranch, the 2nd–4th gill arches with holobranchs. Six small, pointed rakers on third gill arch. The subopercular element is present/absent as the terminal bone posteriorly. Subopercle indistinguishable from opercle, shaped as its posterior elongated extension, not forming or having subopercular spine. Six branchiostegals. Nasal bones hook‐shaped. Maxillary, premaxillary, nasal and ceratobranchial 5 bones shaped as on Figure 5d.

**Etymology.** The Latin adjective masculine singular nominative “piger” in *Lepadogaster piger* Nardo 1827, meaning slow‐moving, was changed to “pigra” in *Gouania pigra* which is an adjective feminine singular nominative, following the necessity of agreement in gender (Article 31.2, ICZN, 1999).

**Ecology and geographical distribution (Figure** **1** **a).** *Gouania pigra* is endemic to the Adriatic Sea and hence the only purely marine endemic fish known for this basin. The southernmost record is from Vlorë (Albany) and it was (so far) not found on Corfu. Quantitative data on ecology is largely lacking. Throughout its distribution range the species occurs in sympatry with *G. adriatica* **sp. nov.** Sympatric and syntopic occurrence with both *G. adriatica* **sp. nov.** and *G. hofrichteri* **sp. nov.** is only known from Pelješac (Figure 1c). The species inhabits intertidal pebble beaches, where it might be found even above the waterline during low tide, and only rarely occurs in fields of larger boulders. See Supporting Information Video S1 for locomotion behaviour.

**Remarks.** *G. pigra* (Nardo 1827) differs from all other *Gouania* species by the posterior angle of jaws extending to, or close to, a vertical line drawn through the anterior edge of the anterior nostril (*vs*. posterior angle of jaws extending to between a vertical line drawn through the posterior edge of the anterior nostril and a vertical line drawn through the anterior edge of the eye in three other *Gouania* species or even to below the anterior edge of the eyes to the anterior part of the eye in *G. orientalis* **sp. nov.**). *G. pigra* differs from all other congeneric species also by its overall reduced pigmentation. *G. pigra* differs from the three stout‐bodied *Gouania* species (*G. adriatica* **sp. nov.**, *G. orientalis* **sp. nov.** and *G. willdenowi*) by dorsal head profile “S” curved, concave above eye and convex at nape (*vs*. dorsal head profile straight between nape above eye and upper lip tip), the absence of star‐like pigmentation around eyes (*vs*. no pigmentation around eyes) and a large number of vertebrae (Supporting Information Table S2; 39–40 *vs*. 35–38). Five morphometric characters as percentages of standard length of *G. pigra* are nonoverlapping in range with all three stout‐bodied *Gouania*: head length, pectoral‐fin length, prepectoral distance, ventral adhesive disc length and caudal fin‐length (values in the Table 1). There are also morphometric characters nonoverlapping in range with one or two out of three stout‐bodied *Gouania* species (Table 1). In addition, it differs from *G. willdenowi* by infraorbital invagination below anterior half of eye or below mideye (*vs*. infraorbital invagination vertical to posterior part of eye); from *G. orientalis* **sp. nov.** by pectoral rays 13–16 (*vs*. pectoral rays 17–19), upper attachment of disc membrane attaching to base of pectoral fin at 12th–15th pectoral ray (*vs*. upper attachment of disc membrane attaching to base of pectoral fin at 16th–18th pectoral ray), infraorbital invagination below anterior half of eye or below mideye (*vs*. infraorbital invagination vertical to posterior part of eye); and from *G. adriatica* **sp. nov.** by posterior opercular edge with two tips, upper longer or equal to lower (*vs*. posterior opercular edge with pointed upper tip and rounded lower edge) and principal caudal rays 10–11 (*vs*. principal caudal‐fin rays 12–13). *Gouania pigra* differs from another slender‐bodied species, *G. hofrichteri* **sp. nov.**, by posterior opercular edge with two tips, upper longer or equal to lower (*vs*. posterior opercular edge with pointed upper tip and rounded lower edge), longitudinal infralateral and suborbital transversal rows of superficial neuromasts placed in the well‐defined deep groove (*vs*. longitudinal infralateral and suborbital transversal rows of superficial neuromasts placed in shallow groove disappearing in posterior part of longitudinal infralateral row), body cross‐section behind pectoral fin base half oval to pentagonal with straight ventral side (*vs*. body cross‐section behind pectoral fin base triangular with ventral flat and dorsal pointed), and the granules on body shallow and inconspicuous (*vs*. granules on body, at least on posterior part and nape, large and prominent). It is known from the Adriatic Sea and has a nonoverlapping geographic distribution range with *G. orientalis* **sp. nov.**, and *G. willdenowi*. Based on the distributional data presented here, differential morphological and genetic characters as well as the taxonomic positioning of the species *G. pigra* (see Discussion, *Taxonomical and systematic considerations* below) and the fact that no original types (holo‐, lectotypes) exist we designate a neotype for this species. The designated neotype was collected on the island of Krk, close to the original locus typicus (Rovinj, northern Adriatic Sea) and is accessible at the voucher numbers PMR VP3529 at the PMR.

**Neotype designation.** We designated a neotype for *G. pigra*, fulfilling the qualifying conditions (Article 75.3, ICZN, 1999). We are positive that no name‐bearing type specimens exist for *G. pigra* (Fricke *et al*., 2020b; Article 75.3.4, ICZN, 1999). The genus *Gouania* represents a complex zoological problem (Article 75.2, ICZN, 1999) of morphologically very similar congenerics. The redescription of *G. willdenowi*, resurrection of *G. pigra* and description of three new species of *Gouania* in the hitherto monotypic genus thus represented an exceptional need for designation of a neotype for *G. pigra* (Article 75.3, ICZN, 1999). The situation could become even more complicated if more *Gouania* linages were to be found around the Mediterranean. In that case, the name‐bearing material of the present species, holotypes and neotypes, should be available for comparison with potential new material. The diagnostic characters are stated in the species redescription (Article 75.3.1, ICZN, 1999), which is sufficient to ensure the recognition of the species (Article 75.3.3, ICZN, 1999). The neotype fits the original species descriptions of *G. pigra* (Nardo, 1827a and Nardo, 1827b; Article 75.3.5, ICZN, 1999) and the neotype were collected close to the original type localities (Article 75.3.6, ICZN, 1999). The neotype is stored in a scientific museum collection (Article 75.3.7, ICZN, 1999).

#### 3.1.6 *Gouania willdenowi* (Risso 1810)

**English name: Blunt‐snouted clingfish**

**FIGURE 9:**
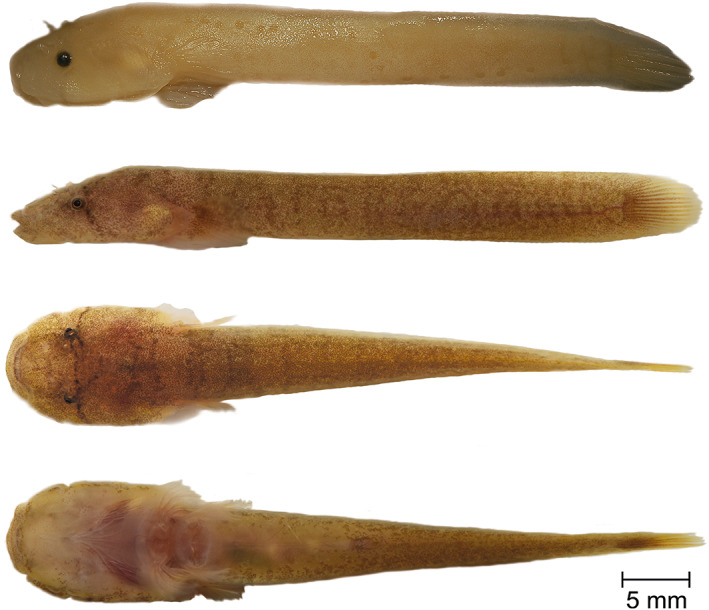
*Gouania willdenowi* (Risso 1810), PMR VP4574, neotype, male, 46.11+6.56 mm, Cagnes‐sur‐mer, Nice, France. Lateral view of specimen preserved in 4% formaldehyde (top). Lateral, dorsal and ventral view, alive (below). Photographs by M. Wagner and M. Kovačić

**Neotype.** PMR VP4574, male, 46.11 + 6.56 mm, Cagnes‐sur‐mer, Nice, France, 43°39′22.1″N, 7°10′25.3″E, coll. M. Wagner, October 9, 2016 (Figure 9).

**Additional material examined.** PMR VP4568, female, 36.2 + 5.18 mm, PMR VP4569, female, 33.65 + 4.93 mm and PMR VP4570, male, 31.13 + 4.36 mm, all from Banyuls‐sur‐mer, France, 42°29′18.6″N, 3°07′43.9″E, coll. M. Wagner, October 11, 2016; ZSM‐PIS‐047654, female, 40.03 + 5.41 mm, Cagnes‐sur‐mer, Nice, France, 43°39′22.1″N, 7°10′25.3″E, coll. M. Wagner, October 9, 2016; PMR VP4575, male, 42.34 + 6.01 mm, PMR VP4576, female, 40.5 + 5.58 mm and ZSM‐PIS‐047655, male, 28.42 + 5.94 mm, all from Cagnes‐sur‐mer, Nice, France, 43°39′22.1″N, 7°10′25.3″E, coll. M. Wagner, October 10, 2016; PMR VP4577, female, 39.59 + 5.63 mm and PMR VP4578, female, 36.82 + 5.4 mm, both from Le Port d'Alon, Toulon, France, 43°08′47.8″N, 5°42′27.2″E, coll. M. Wagner, October 16, 2016.

**Synonyms.** *Lepadogaster willdenowi* Risso, 1810: 75 (original description; type locality: Nice; holotype: unknown); *Rupisuga nicensis* Swainson 1839: 339 (original description; type locality: Nice?; holotype: unknown), *Lepadogaster latirostris* Costa 1850: 4 (original description; type locality: Naples; holotype: unknown); *Leptopterygius wildenowi* Troschel, 1860: 206 (original description; type locality: Nice; holotype: unknown); *Leptopterygius coccoi* Troschel, 1860: 207 (original description; type locality: Messina; holotype: unknown).

**Diagnosis.** *Gouania willdenowi* differs from congeneric species by the combination of the following characters: (1) dorsal head profile straight between nape above eye and upper lip tip; (2) posterior angle of jaws extends to between a vertical line drawn through the posterior edge of the anterior nostril and a vertical line drawn through the anterior edge of the eye; (3) infraorbital invagination vertical to posterior part of eye; (4) posterior opercular edge w‐shaped with two equally long tips; (5) longitudinal infralateral and suborbital transversal rows of superficial neuromasts placed in the well‐defined deep groove; (6) trunk cross‐section behind pectoral fin base half oval with straight ventral side; (7) granules on body shallow and inconspicuous; (8) vertical eye diameter 2.6–3.3% of standard length; (9) horizontal eye diameter 2.3–2.9% of standard length; (10) head length 24.3–28.8% of standard length; (11) pectoral‐fin length 8.6–9.3% of standard length; (12) prepectoral distance 24.4–28.4% of standard length; (13) ventral adhesive disc length 17.1–19.3% of standard length; (14) caudal‐fin length 13.5–15.5% of standard length; (15) moderate number of vertebrae for the genus (Supporting Information Table S2; 37–38); (16) pharyngeal jaws with small ceratobranchial 5, having several small conical teeth; (17) nasal bones club‐shaped; (18) star‐like pigmentation around eyes, in life body colouration dark with clear stripes visible in less pigmented specimens; (19) distribution range restricted to the western Mediterranean.

**Description.** *General morphology*: Body proportions are given in Table 1. Body slender and elongated, posteriorly laterally compressed, body depth at pectoral fins 7.5–8.8 in SL, body depth at anus 8.8–11.4 in SL, body depth in width at pectoral fins 1.1–1.3, body depth in width at anus 0.8–0.9. Body cross‐section behind pectoral fin base half oval with straight ventral side. Granules on body shallow and inconspicuous. Head dorsoventrally compressed, head depth in width at orbit 1.5–1.9, and moderately large, head length 3.5–4.1 in SL, head wider than body width maximum, head width at anterior sucking disc edge 0.7–0.8 in body width at pectoral fins. Dorsal head profile straight between nape above eye and upper lip tip. Head rounded in dorsal view. Snout large compared to eyes, preorbital distance 2.8–3.5 in head length, 0.3 in horizontal eye diameter. Snout wide, not produced, blunt. Internostril space gently convex. Eyes dorsolateral, with lower eye edge rounded. Eyes small, 9.1–11.0 in head length, vertical diameter of the eye 0.8–1.0 in horizontal eye diameter. Infraorbital invagination vertical to posterior part of eye. Interorbital distance wide, 0.3–0.4 in horizontal eye diameter. Centre of eye much closer to tip of snout than to posterior margin of operculum, preorbital distance in postorbital distance 1.7–2.3. Anterior and posterior nostrils long tubes of about equal length. Nostrils well separated and posterior nostril located behind and dorsally to the anterior edge of eyes. Single large dermal flap of leaf shape at the posterior margin of anterior nostril, longer than nostril. Posterior nostril rim crenate with no extension. Skin with small granules. Head lateral line system with canals with pores and with superficial neuromasts arranged in rows. Head canals reduced and pores small. Single pore in nasal canal near posterior nostril. Single pore in postorbital canal close to posterior eye edge. Two pores in mandibular canal, anterior one close to anteriolateral angle of mouth, posterior pore slightly in front of vertical of posterior angle of jaws, posterior pore usually more prominent. Lachrymal as well as preopercular canals and pores absent. Rows of superficial neuromasts as follows: SR 2, NR 3, LIR 24–28, STR 3–4, POR 3–4, PTR 2–3, SLR 5–6, MR 10–11, AVR 2, PVR 1, ADR 2–3, PDR 0–1, HR 3–4, SR1 3–4, SR2 1–2, DLR 6–9, VLR 11–14. STR and LIR rows of superficial neuromasts placed in the well‐defined deep groove. DLR row of superficial neuromasts anteriorly starts above pectoral fin, continuously dorsolateral and ends posteriorly downwards at midlateral level above anus. VLR anteriorly starts behindpectoral fin base, continuous ventrolaterally and ends posteriorly upwards with last papilla nearly at midlateral level at caudal‐fin base. Mouth terminal, upper and lower lips ends about equally, lips fleshy, upper lip larger than the lower lip. Posterior angle of jaws extends to between vertical line drawn through posterior edge of anterior nostril and vertical line drawn through anterior edge of eye. Chin with bilobed fold at anterior edge covering MR row of superficial neuromasts. The gill membrane is attached to isthmus, gill opening starting at the base of pectoral fin, with the upper attachment of the gill membrane is opposite to 4th–6th pectoral ray. Posterior opercular edge w‐shaped with two equally long tips. No subopercular spine. No fleshy pad present on lower pectoral base. Urogenital papilla present. Preanus length in postanus length 0.7–0.8. Anal papillae absent, the area around anus wrinkled.

*Fins*. Rudimentary dorsal and anal fins located well posteriorly and short, reduced to low ridges with very weak rays, connected to the caudal fin. Pectoral rays 16–19. Caudal fin rounded, principal caudal rays 11–12. Ventral adhesive disc (Figure 4e) of “double” type, anterior margin crenate with large invagination on each lateral side; posterior margin crenate. Disc small, disc length 5.2–5.9 in SL, its width slightly larger than its length, width in length 0.9–1.0. No papillae in region A and flattened papillae in regions B and C. In region B two rows of papillae with total papillae count 17–28 and in region C two rows of papillae with total papillae count 9–14. No inner row of papillae on lateral sides of the central part of the anterior disc. Upper attachment of disc membrane attaching to base of pectoral fin at 15th–18th pectoral ray, *i.e*., penultimate ray. Males can have two prominent seemingly perfused finger‐like extensions on each site of sucking disc that sometimes equal or exceed length of disc region A (Figure 4f) (Hofrichter, 1995; Hofrichter & Patzner, 2000).

*Colouration*. Background colouration of live specimens flesh‐coloured, orange or yellow (Figure 9) and star‐like pigmentation around eyes present. In life body coloration with dark clear stripes visible in less pigmented specimens (*e.g*., Messina), other specimens with irregular marbled pattern. Formaldehyde fixed specimens white‐yellow and without pigments. Ethanol fixed specimens white to skin coloured, striped pigmentation visible. For more pictures of life colouration see Supporting Information File S1.

*Dentition and osteology*. Upper jaw with outer row four (one side) medium‐sized caniniforms frontally, two median teeth larger. Behind them inner small conical teeth irregularly scattered in two separate (left and right) droplike patches medially wide about 4–5 teeth, becoming narrowed to a single row of teeth laterally. Outer row continues laterally as single large caniniform, followed behind by three medium‐sized caniniforms. Lower jaw with outer row of eight–10 (one side) medium‐sized caniniforms frontally. Behind them single broad patch of small conical inner teeth medially wide about 3–4 teeth, becoming narrowed to a single row of teeth laterally. The single row of four larger caniniforms continuous laterally. Pharyngeal jaws with small ceratobranchial 5 having several (6–7) small conical teeth (Figure 5e), pharyngobranchial 3 toothplate not visible on 3D models from microcomputed tomography (microCT) images. Number of vertebrae 37–38, abdominal 16 and caudal 21–22 (Supporting Information Table S2). The first gill arch with hemibranch, the 2nd–4th gill arches with holobranchs. Subopercle indistinguishable from opercle, shaped as its posterior elongated extension, not forming or having subopercular spine. Six branchiostegals. Club‐shaped nasal bones. Maxillary, premaxillary, nasal and ceratobranchial 5 bones shaped as on Figure 5e.

**Etymology.** Risso (1810) formed the name from a personal name, as the noun in the genitive case, with “i” added to the stem of the name (presently under Article 31.1.2., ICZN, 1999). We followed the spelling of the species as recommended by Fricke *et al*. (2020b), “*G. willdenowi”*. In the text of the original description it appeared as “4. L. Willdenow. N. *L. Willdenowi*”. So we concluded that “*Wildenowii”* in the index represents a case of misspelling (Risso, 1810). We think that the name was given in honour of Carl Ludwig Willdenow, even though this is not explicitely stated in Risso (1810). Hence, “*L. willdenowi”* would be the correct spelling of the name.

**Ecology and geographical distribution (Figure** **1** **a).** The distribution range of *G. willdenowi* is restricted to the western Mediterranean basin with an easternmost record from Messina (Sicily). The species reaches highest abundances in pebble and boulder beaches of less than 0.5 m depth (24 individuals/m^2^ in Messina), but can be found down to 2 m of water depth (Hofrichter & Patzner, 2000; Patzner, 1999). *Gouania willdenowi* can be sometimes found in sympatry with *Lepadogaster lepadogaster* and males build and guard nests under boulders during spawning season (Hofrichter, 1995; own observations).

**Remarks.** *Gouania willdenowi* differs from known congeners by various characters among which the most useful were selected for diagnosis and are elaborated on here. Some of the characters differing among (some) species and that are not used in the species diagnosis are nonetheless mentioned here as they are interesting for comparison. *G. willdenowi* differs from slender‐bodied *Gouania* species (*G. pigra* and *G. hofrichteri* **sp. nov.**) by a straight dorsal head profile between nape above eye and upper lip tip (*vs*. dorsal head profile in lateral view “S” curved, concave above eye and convex at nape), infraorbital invagination vertical to posterior part of eye (*vs*. infraorbital invagination below anterior half of eye or below mideye) and a star‐like pigmentation around eyes (*vs*. no pigmentation around eyes). Eleven morphometric characters as percentages of standard length of *G. willdenowi* are nonoverlapping in range with both slender‐bodied *Gouania*: head length, all three head width measures, preorbital distance, pectoral‐fin length, prepectoral distance, ventral adhesive disc length, distance between the posterior margin of sucking disc and anus, caudal base depth and caudal‐fin length (values in the Table 1). There are also morphometric characters nonoverlapping in range with only one of the two slender‐bodied *Gouania* (Table 1). In addition, *G. willdenowi* differs from *G. pigra* by a posterior angle of jaws which extending to between vertical line drawn through posterior edge of anterior nostril and vertical line drawn through anterior edge of eye (*vs*. posterior angle of jaws extending at, or close to, vertical line drawn through anterior edge of anterior nostril). *G. willdenowi* also differs from *G. hofrichteri* **sp. nov.** by posterior opercular edge w‐shaped with two equally long tips (*vs*. posterior opercular edge with pointed upper tip and rounded lower edge), longitudinal infralateral and suborbital transversal rows of superficial neuromasts placed in the well‐defined deep groove (*vs*. longitudinal infralateral and suborbital transversal rows of superficial neuromasts placed in shallow groove disappearing in posterior part of longitudinal infralateral row), body cross‐section behind pectoral fin base half oval with straight ventral side (*vs*. trunk cross‐section behind pectoral fin base triangular with ventral flat and dorsal pointed) and the granules on body shallow and inconspicuous (*vs*. granules on body, at least on posterior part and nape, large and prominent). *G. willdenowi* differs from the stout‐bodied species *G. adriatica* **sp. nov.** by posterior opercular edge w‐shaped with two equally long tips (*vs*. posterior opercular edge with pointed upper tip and rounded lower edge) and by vertical eye diameter 2.6–3.3% and horizontal eye diameter 2.3–2.9% of standard length (*vs*. vertical eye diameter 3.4–4.3% and horizontal eye diameter 3.0–3.7% of standard length). *G. willdenowi* has no nonoverlapping external morphological differences to *G. orientalis* **sp. nov.** but differs by its larger number of vertebrae (Supporting Information Table S2; 37–38 *vs*. 35–36). *G. willdenowi* is only known from the Western Mediterranean and has nonoverlapping geographic distribution with all other *Gouania* species. Based on the distributional data presented here, morphological and genetic data as well as the taxonomic positioning of the species *G. willdenowi* (see Discussion, *Taxonomical and systematic considerations* below) and the fact that no original types (holo‐, lectotypes) exist we designate a neotype for this species. The designated neotype was collected close to the original locus typicus (Nice, France) and is accessible as the voucher number PMR VP4574 at the PMR.

**Neotype designation.** We designated a neotype for *G. willdenowi*, fulfilling the qualifying conditions (Article 75.3, ICZN, 1999). We are positive that no name‐bearing type specimens exist for *G. willdenowi* (Fricke *et al*., 2020b; Article 75.3.4, ICZN, 1999). The genus *Gouania* represents a complex zoological problem (Article 75.2, ICZN, 1999) of morphologically very similar congenerics. The redescription of *G. willdenowi*, resurrection of *G. pigra* and description of three new species of *Gouania* in the hitherto monotypic genus thus represented an exceptional need for designation of a neotype for *G. willdenowi* (Article 75.3, ICZN, 1999). The situation could become even more complicated if more *Gouania* linages were to be found around the Mediterranean, which is not at all unlikely. In that case, the name‐bearing material of the present species, holotypes and neotypes, should be available for comparison with potential new material. The diagnostic characters are stated in the species redescription (Article 75.3.1, ICZN, 1999), which is sufficient to ensure the recognition of the species (Article 75.3.3, ICZN, 1999). The neotype fits the original species descriptions of *G. willdenowi* (Risso, 1810; Article 75.3.5, ICZN, 1999) and the neotype were collected close to the original type localities (Article 75.3.6, ICZN, 1999). The neotype is stored in a scientific museum collection (Article 75.3.7, ICZN, 1999).

### 3.2 Genetics

**FIGURE 10:**
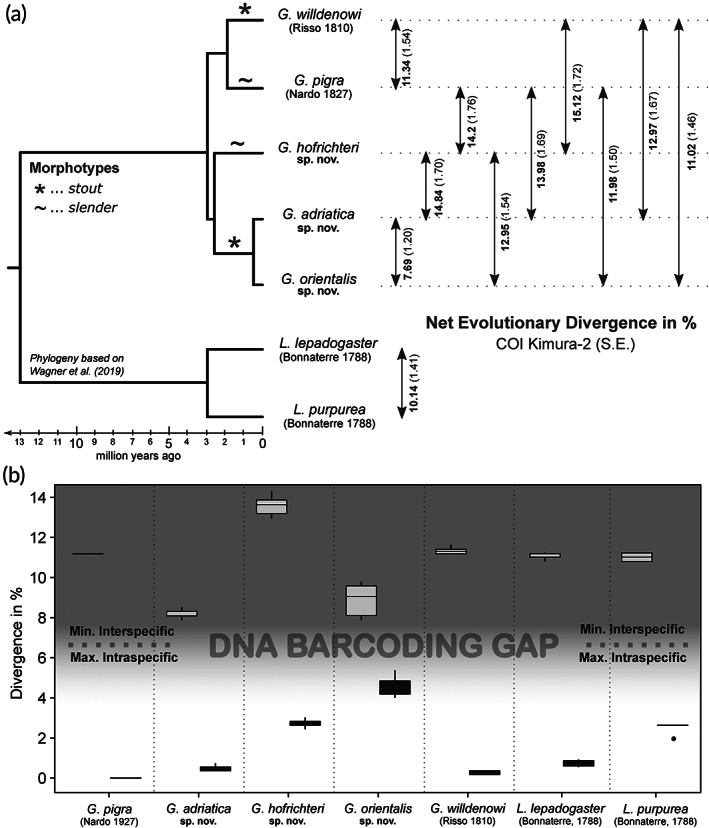
Results of DNA‐barcoding analyses. (a) Net between‐group mean distances (mean and S.E.). The phylogeny shown in this figure is based on Wagner *et al*. (2019). (b) DNA‐barcoding gap represented by boxplots showing maximum intraspecific and minimum interspecific divergences in %

Based on COI sequence data, the genus *Gouania* can be divided into five major lineages with net divergences (Kimura‐2 paramter model) between groups ranging from 7.69% to 15.12% (Figure 10a). These genetic clusters are also supported by previous multilocus phylogenetic analyses (Wagner *et al*., 2019). Using clingfish specific substitution rates following Conway *et al*. (2017a), the onset of the *Gouania* radiation was dated to 3.18 (95% highes posterior density interval, 2.06–5.57) million years ago (Wagner *et al*., 2019). Furthermore, this phylogeny places *G. orientalis* **sp. nov.** as the sister group of *G. adriatica* **sp. nov.**. The investigation of minimum interspecific *versus* maximum intraspecific divergence (in %) revealed a clear gap (“barcoding gap”) between these two values for all the *Gouania* species (Figure 10b). Nonetheless, we found high maximum intraspecific values for the eastern Mediterranean clades *G. hofrichteri* **sp. nov.** (2.44–3.02%) and even larger ones for *G. orientalis* **sp. nov.** (4.00–5.38%), which suggests strong phylogeographic/population genetic structure within these clades.

## 4 DISCUSSION

### 4.1 Taxonomic considerations

In this work, based on genetic, morphological and biogeographic evidence, and building upon our previous phylogenetic study (Wagner *et al*., 2019), we describe three new species of the genus *Gouania* (Nardo 1833) for the Mediterranean Sea and provide redescriptions and designation of neotypes for *G. willdenowi* and *G. pigra*. Generally, the systematics and taxonomy of European clingfishes is complex and highly influenced by descriptions of the 19th century (Briggs, 1955; Hofrichter, 1995). This is also true for *Gouania*, with the first uncertainties arising already with the description of *G. willdenowi*. Risso (1810) delivered a very poor drawing alongside a partly fanciful description (due to the typical writing style in those days), which was later criticized by many authors (Canestrini, 1864; De Filippe, 1861; Troschel, 1860) and could be responsible for the overall taxonomic confusion in the genus (Hofrichter, 1995). Because Risso's description was not discriminative for any of our five species of *Gouania* and because the type material of the original description is not available anymore (Fricke *et al*., 2020a,b), we designated the only *Gouania* species occurring at the original type locality (Nice), and the western Mediterranean basin, as *G. willdenowi* (with a neotype), according to Article 75.3 of the ICZN. Briggs (1955) and Hofrichter (1995) listed several synonyms, four of which, *Rupisuga nicensis* Swainson1839, *Lepadogaster latirostris* Costa 1850, *Leptopterygius wildenowi* Troschel 1860 and *Leptopterygius coccoi* Troschel 1860, can be geographically and/or morphologically linked to *G. willdenowi*.

Whereas no species were described from the eastern Mediterranean basin, two further *Gouania* descriptions from the Adriatic Sea (Rovinj) are known, *Lepadogaster piger* Nardo 1827 (Nardo, 1827a, which he repeated in Nardo, 1827b), and *Gouania prototypus* Nardo 1833. In general, Nardo's descriptions are more accurate than the one by Risso (1810), but there is evidence that *G. prototypus* is a junior synonym of *G. pigra*. In Nardo's original description of *Gouania* he directly refers twice to the species *L. piger* (Nardo, 1833: 548: „(…) *Eine neue Art von Lepadogaster*, *welcher Rücken und Afterflosse fehlen*, *und die er Lepadogaster piger nennt*. (…) *Lepadogaster piger Nardo Prod*. (…), *hat ein Merkmal*, *welches wichtig genug ißt*, *um sie zu einer eigenen Sippe zu erheben*, *welche er Covania* (…), *nennen will mit dem Spezies Namen prototypus*”), which he elevates to the new genus and gives (for unknowable reasons) a new species epithet *prototypus*. The fact that Nardo (1860:79) himself only mentions *G. pigra* in his later work and Steindachner (1868:687), listed *G. pigra* s. *prototypus* Nardo as a synonym of *G. willdenowi* supports this hypothesis. We therefore regard *prototypus* as a synonym of *pigra*. Due to a change in the genus name (from *Lepadogaster* to *Gouania*), in accordance with Article 31.2, ICZN, 1999, the species epithet *piger* was changed to *pigra*, to avoid misspelling. The putative locus typicus of *G. pigra* (Rovinj, northern Adriatic Sea) as well as all characters mentioned in Nardo's descriptions (Nardo, 1827a:102, Nardo, 1833:548) would match, except for the pectoral fin ray count, both Adriatic species, “Adriatic slender” and “Adriatic stout” (according to the terminology of Wagner *et al*., 2019). In the description, Nardo (1833:548) mentions a total pectoral fin ray count of 12 for *G. pigra* s. *prototypus*. Albeit not matching the fin ray count of either species, this is quite far from the known “Adriatic stout” pectoral ray count, which is 15–17, and the most parsimonious explanation is that these pectoral counts are not present in this species. Our “Adriatic slender” specimens, however, have a pectoral ray count of 13–16, which might indicate that either Nardo miscounted pectoral rays by one ray or that the true range in *G. pigra* population for the pectoral fin ray count is indeed 12–16, slightly larger than established on the present material. Therefore, we assigned the “Adriatic slender” morphotype presented in Wagner *et al*. (2019) to *G. pigra* and designated a neotype from the type locality. The taxonomic revision of *Gouania*, including a single valid species (*G. willdenowi*), one resurrected species (*G. pigra*) and three newly described species (*G. adriatica*, *G. orientalis* and *G. hofrichteri*), forms the necessary foundation for further biological, ecological and evolutionary investigations of this genus.

### 4.2 The *Gouania* radiation in the Mediterranean Sea: an eco‐evolutionary outlook

Tiny cryptic fishes, or in a broader sense cryptobenthic fishes, are among the most diverse groups of vertebrates on this planet, which can be linked to their biology and ecology (reviewed by Brandl *et al*., 2018). First, their small body size enables them to invade a variety of niches, inaccessible for large reef fishes, and allows coexisting competing species to partition niches between them (*e.g*., Ahmadia *et al*., 2018; Brandl *et al*., 2020; Goatley *et al*., 2016; Herler, 2007; Kovačić *et al*., 2012; Rüber *et al*., 2003; Tornabene *et al*., 2013). Second, due to their rather stationary benthic lifestyle and limited active dispersal abilities, they are particularly prone to diversify in the face of spatial reproductive isolation (*e.g*., Colin, 2010; Tornabene *et al*., 2015; Winterbottom *et al*., 2014).

This is also true for the genus *Gouania*, which inhabits the narrow interstitial space of intertidal pebble beaches (Hofrichter & Patzner, 2000). Beyond their small size (typically <50 mm SL), they evolved remarkable morphological and behavioural adaptations that foster their survival in an otherwise hostile environment. Hence, *Gouania* has the largest number of vertebrae compared to all other European clingfishes (Briggs, 1955), which promotes an increased body flexibility and could be the decisive evolutionary factor for invading this particular habitat (Wagner *et al*., 2019; Yamada *et al*., 2009). Furthermore, studies showed that, during low tide, *Gouania* can endure for hours in moist parts of pebble layers without being harmed (Bilecenoğlu, 2015). This amphibious emergence behaviour is considered passive rather than active and is most likely linked to tidal changes (Hofrichter & Patzner, 2000; Bilecenoğlu, 2015; own observations). Yet, general knowledge about biology, ecology or behaviour of the genus *Gouania* remains scarce. Compared to numerous studies that have been conducted on the sister genus *Lepadogaster* (*e.g*., Faria & Gonçalves, 2010; Gonçalves *et al*., 1996, 1998; Hofrichter & Patzner, 2000; Tojeira *et al*., 2012; Trkov & Lipej, 2019), only a few ecological studies mention *Gouania* (Hofrichter, 1995; Hofrichter & Patzner, 2000; Kovačić, 1997; Patzner, 1999). Apart from the studies performed in the western Mediterranean, which deliver important insights into the microhabitat characteristics of *G. willdenowi*, it is impossible to relate these data to any of the (re)described *Gouania* species. Nonetheless, the taxonomic revision of the genus *Gouania* opens up new opportunities to investigate putative ecological aspects that triggered macroevolutionary changes in the *Gouania* radiation. Niche partitioning is a crucial driver in cryptobenthic fish diversification (Brandl *et al*., 2018) and could be a key factor for explaining the independent evolution of sympatrically occurring *Gouania* morphotypes “stout” (*G. willdenowi*, *G. adriatica* **sp. nov.** & *G. orientalis* **sp. nov.**) and “slender” (*G. pigra*, *G. hofrichteri* **sp. nov.**) in the Adriatic and eastern Mediterranean basin. We discussed previously that due to their increased number of vertebrae (>38), the “slender” morphs could be better adapted to a life in finer gravel than the congeneric “stout” species (Wagner *et al*., 2019). This association between increased body flexibility and microhabitat choice has been previously reported for interstitial gobies (Yamada *et al*., 2009). So far, few quantitative studies have been conducted on Mediterranean cryptobenthic fish assemblages in general (*e.g*., Glavičić *et al*., 2016, 2020; Kovačić *et al*., 2012; Santin & Willis, 2007; Thiriet *et al*., 2016) and knowledge about basic life history traits (*e.g*., reproductive biology, larval behaviour and ontogeny) is lacking for many species. Considering the crucial global role of cryptobenthic fishes for near‐shore ecosystem functioning (Brandl *et al*., 2019; Depczynski & Bellwood, 2003), however, such data are invaluable, particularly because these traits are often linked to population connectivity and diversification patterns in many marine littoral species (*e.g*., Ahmadia *et al*., 2018; Galarza *et al*., 2009; Palumbi, 1994; Riginos *et al*., 2011).

In accordance with their stationary biology (*i.e*., benthic breeding, cryptic behaviour, poor swimming), most cryptobenthic fishes have limited dispersal abilities as adults and the extent of dispersion depends on a temporary pelagic larval phase, which is often used as a proxy for estimating marine population connectivity (reviewed by Selkoe & Toonen, 2011). Since the pelagic larval duration (PLD) is comparatively short in cryptobenthic fishes (Beldade *et al*., 2007; Macpherson & Raventos, 2006), it is assumed that the majority of these taxa show a fine‐scale geographic structure. Indeed, cryptobenthic taxa, including clingfishes, comprise many endemic species and genera (Briggs, 1955; Conway *et al*., 2017c). This is particularly true for the genus *Gouania*, which is endemic to the Mediterranean ecoregion and includes species that are exclusively confined to main basins (Figure 1a). Among truly marine Mediterranean fishes, the small geographic distribution ranges of the various *Gouania* species are considered to be exceptional, especially compared to the sister genus *Lepadogaster*, which shows, despite its much wider distribution range (north‐eastern Atlantic to Mediterranean), almost no phylogeographic structure in the Mediterranean basin (Wagner *et al*., 2017). Additionally, the high values of intraspecific divergences (Figure 10b) in the eastern Mediterranean species, *G. orientalis* **sp. nov.** and *G. hofrichteri* **sp. nov.**, suggest further population substructuring on an even smaller scale. Oceanic currents and fronts (Figure 1a) have been shown to play an important role for shaping population differentiation in several benthic Mediterranean fishes (*e.g*., Galarza *et al*., 2009; Schunter *et al*., 2011; Koblmüller *et al*., 2015; Sefc *et al*., 2020) and also in Gobiesocidae (Klein *et al*., 2016). However, to what extent this is true for cryptobenthic fishes in general and clingfishes in particular remains questionable, since most larvae and juveniles stay nearshore (Beldade *et al*., 2006; Brandl *et al*., 2019; Macpherson & Raventos, 2006; Sefc *et al*., 2020). Larvae of *Gouania* are considered to drift close to shores and unlikely to transcend into larger circulation systems, which would be crucial for long distance dispersal (Macpherson & Raventos, 2006; Wagner *et al*., 2019). Thus, population patterns in *Gouania* might be rather linked to local (*e.g*., winds) and temporal (*e.g*., seasons) factors, similar to the sister genus *Lepadogaster* (Klein *et al*., 2016). Furthermore, studies showed that even congeneric species of *Lepadogaster* can have a different pace and pattern of larval development, which impacts the duration of the planktonic larval stage (Faria & Gonçalves, 2010; Tojeira *et al*., 2012). Warm temperate seas, like the Mediterranean, seasonally fluctuate in water temperature and local current structure, which could impact the development and distribution of *Gouania* larvae (Figure 1a; El‐Geziry & Bryden, 2010). Nonetheless, active behaviour of larvae could influence population differentiation in *Gouania*. Keeping recruits close to natal sites is common among cryptobenthic fishes (Milá *et al*., 2017; Rüber *et al*., 2003) and could be, especially in remote areas (*e.g*., islands), an effective strategy for sustaining populations (Brandl *et al*., 2019). Thus far, ontogenetic studies on *Gouania* are lacking, but studies on *Lepadogaster* clearly showed that after hatching larvae are already very well developed and good swimmers (Faria & Gonçalves, 2010; Guitel, 1888; Tojeira *et al*., 2012), and juveniles of *Apletodon* are able to actively seek suitable microhabitats when they switch to the benthic lifestyle (Gonçalves *et al*., 2002). Extrapolating from this, it is very likely that *Gouania* larvae are already equipped with sensory organs that could allow them to actively return to natal sites (Gerlach *et al*., 2007). Suitable microhabitats for *Gouania* – intertidal pebble beaches – are rare compared to long stretches of bedrock coast at tidal level, resulting in a dotted presence of this habitat (*e.g*., all along the Eastern Adriatic Sea). This additionally increases the need for an active return to natal sites for *Gouania* species, as compared to other clingfish species that typically occur somewhat deeper than *Gouania*. Altogether, this could explain the micro‐allopatric patterns observed in the *Gouania* radiation (also see Wagner *et al*., 2019) and raises the question of whether there is even more unexplored diversity in the genus, particularly in remote areas and locations where *Gouania* was recorded in the past (see records of Hofrichter (1995) in Figure 1a).

To sum up, the *Gouania* radiation is most likely a product of biogeographic and ecological factors and bears high potential for future research. Future studies on the sympatric species pairs of the Adriatic and eastern Mediterranean will unravel the role of adaptive ecological drivers in the early stages of the radiation. Additionally, population genetic studies in combination with oceanographic modelling might illuminate thrilling microevolutionary patterns and more hidden diversity in this enigmatic fish radiation.

## CONTRIBUTIONS

M.W. was responsible for the study design, field work, fish identification, generation and analysis of the data, and wrote the manuscript. M.K. participated in some field work, generated and analysed morphological/morphometric data and contributed to manuscript writing. S.K. participated in some fieldwork, supervised the research and data interpretation and contributed to manuscript writing. All authors read the manuscript and approved the final version.

## Supporting information


**Supporting Information File S1.** Intraspecific colour differences

**Supporting Information Table S1.** Investigated sites, coordinates and *Gouania* species occurrences

**Supporting Information Table S2.** Investigated museum vouchers

**Supporting Information Video S1.** *Gouania pigra* **sp. nov.** in the aquarium
