# Supplementary material for: Unravelling the taxonomy of an interstitial fish radiation: Three new species of Gouania (Teleostei: Gobiesocidae) from the Mediterranean Sea and redescriptions of G. willdenowi and G. pigra
Source: J Fish Biol. 2020 Nov 3;98(1):64–88. doi: 10.1111/jfb.14558 (PMC7821206; doi:10.1111/jfb.14558)
Supplement: Supplementary file 1 — Supporting Information File S1. Intraspecific colour differences [file JFB-98-64-s001.pdf]

Supplementary File 1 Intraspecific color differences

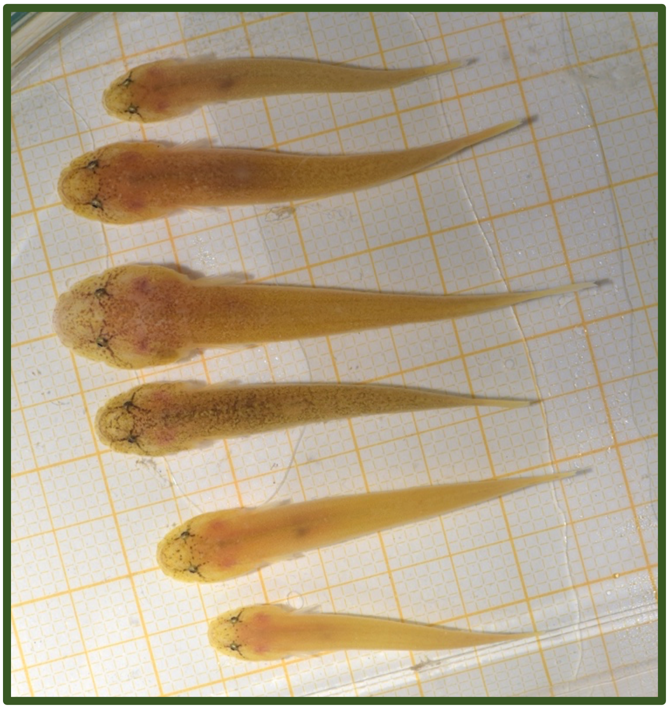

G. adriatica **sp. nov.**
Agni Beach 39.736889, 19.929861

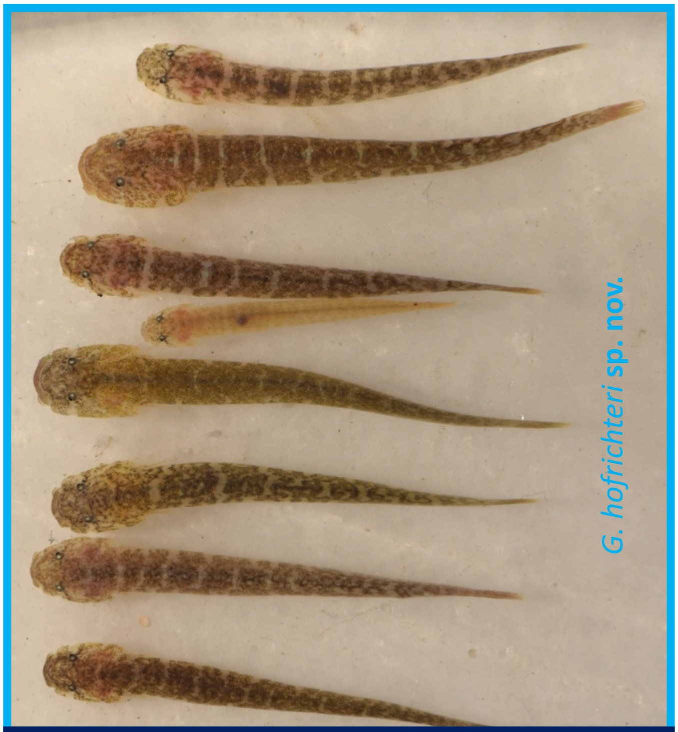

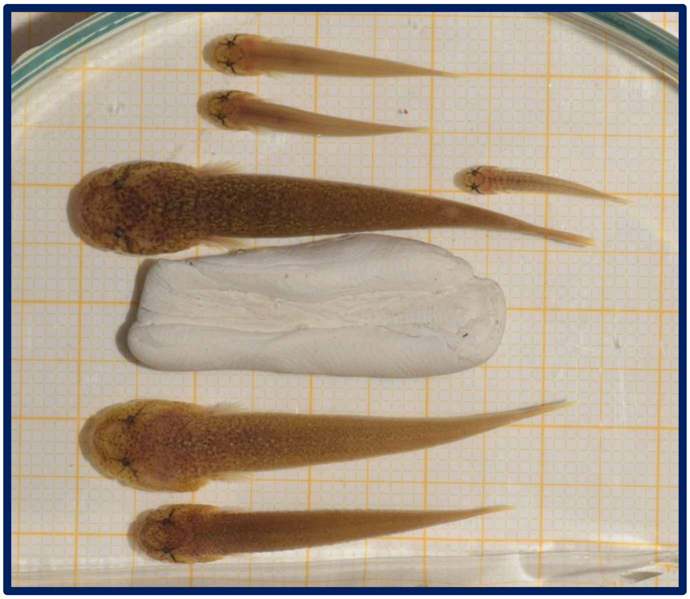

G. orientalis **sp. nov.**
Gulf of Korinth 38.171389, 22.274083

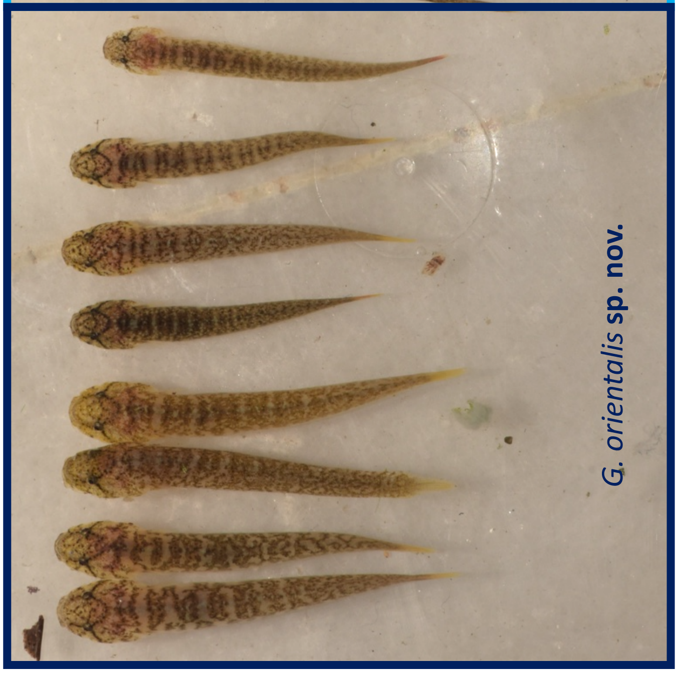

Chamolia 37.916250, 24.035750

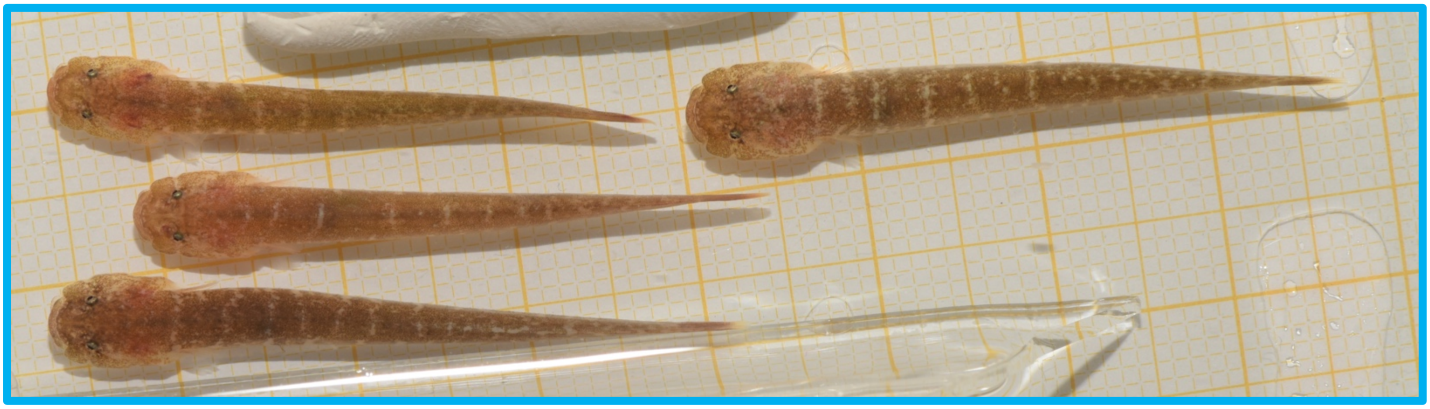

G. hofrichteri **sp. nov. Gulf of Korinth 38.171389, 22.274083**

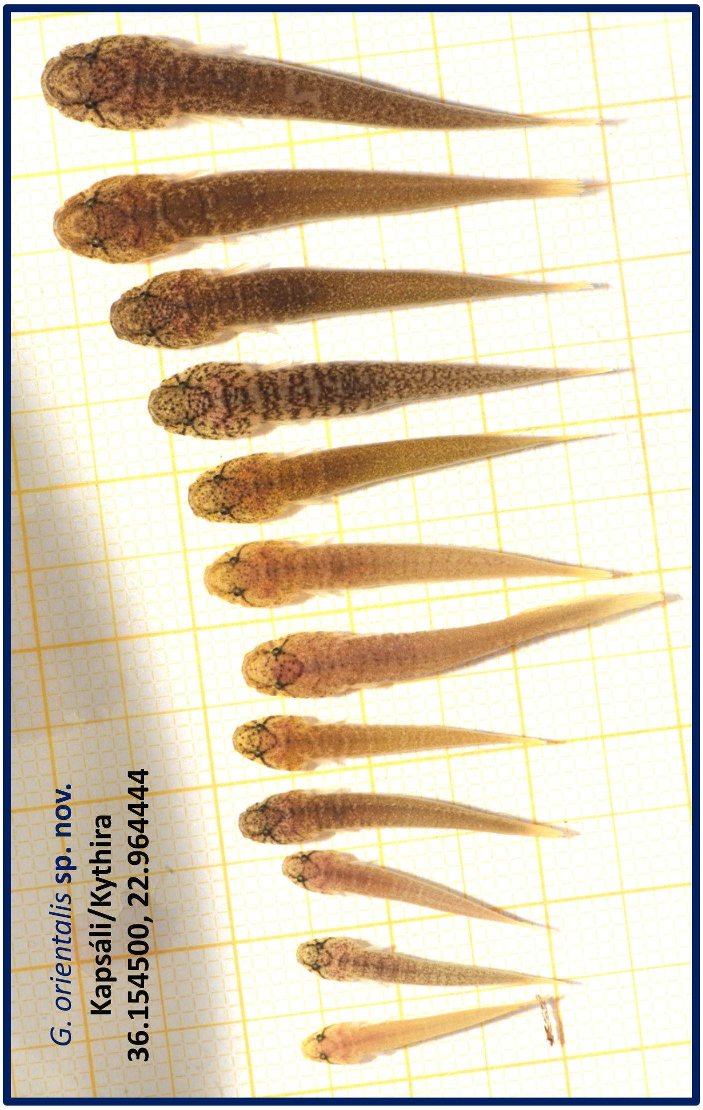

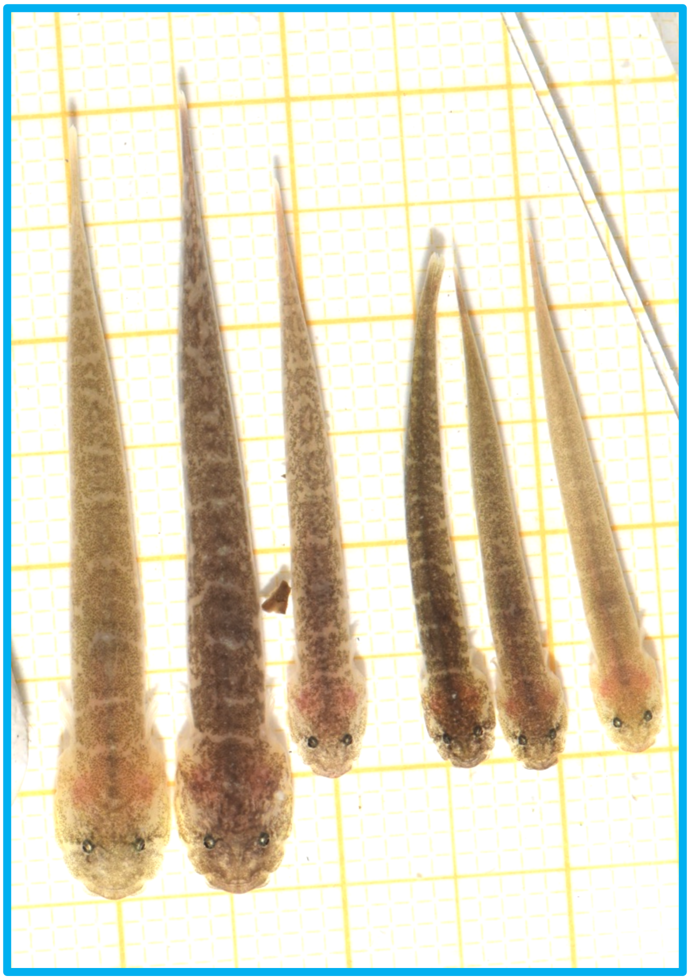

G. hofrichteri **sp. nov.**
Kapsáli/Kythira
36.154500, 22.964444

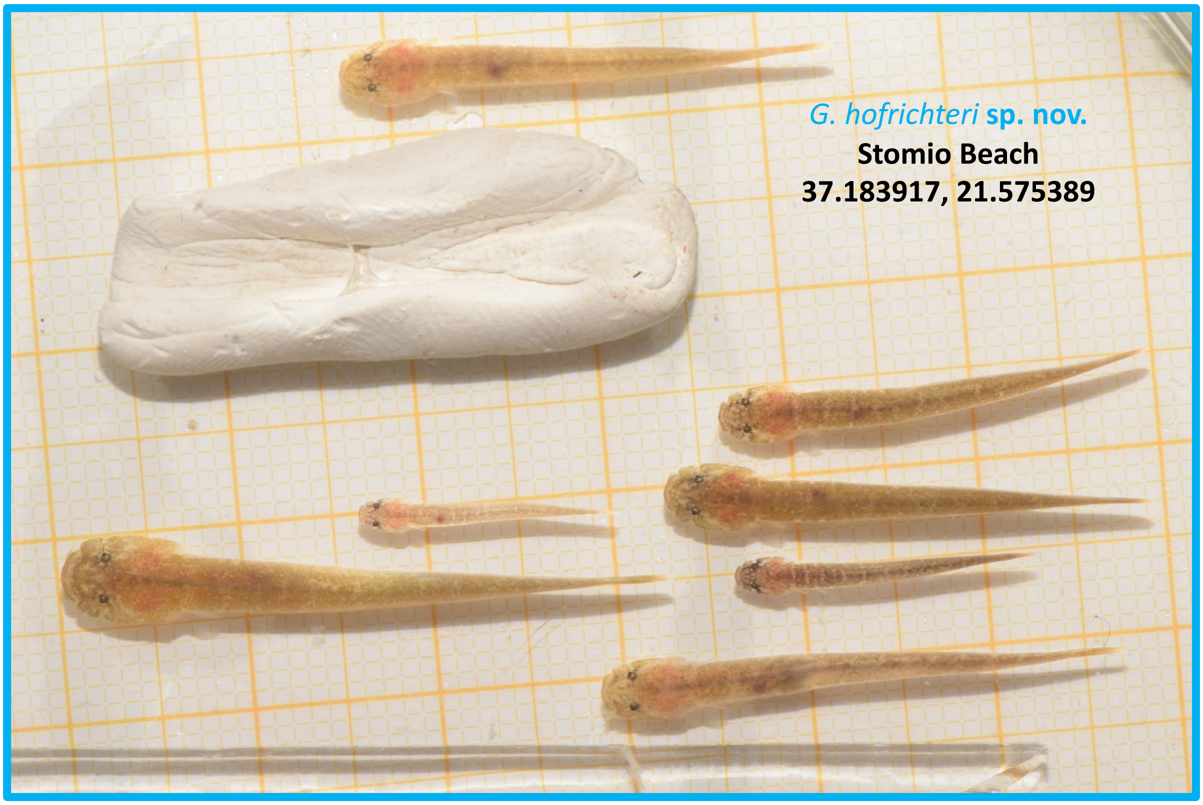

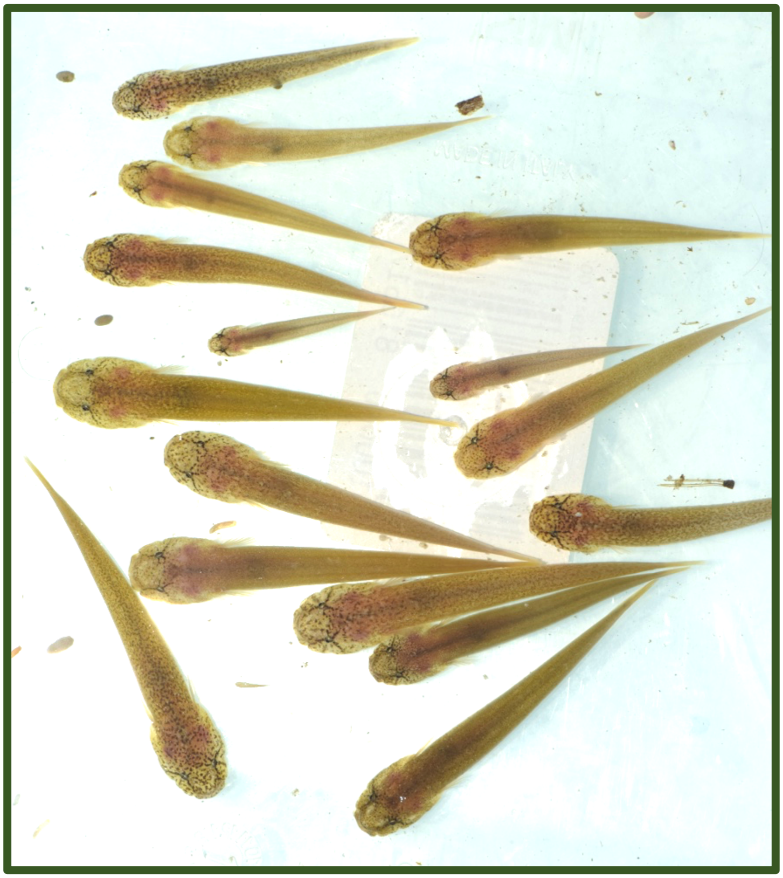

G. adriatica **sp. nov.**
Vlorë 40.387862, 19.482819

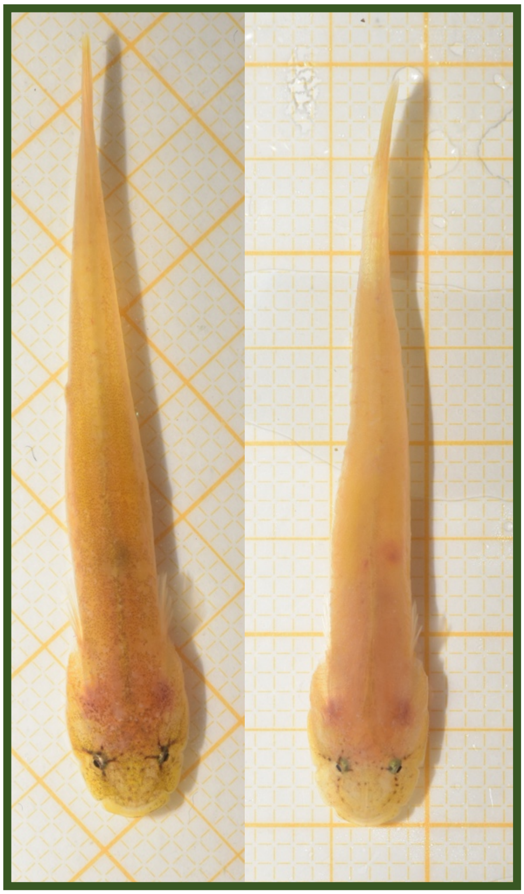

G. adriatica **sp. nov.**
Rijeka 45.314557, 14.469908

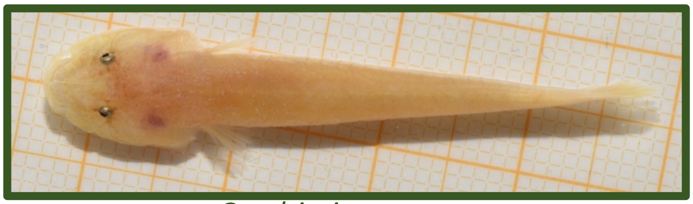

G. adriatica **sp. nov.**
Sv. Marina 45.028361, 14.154833

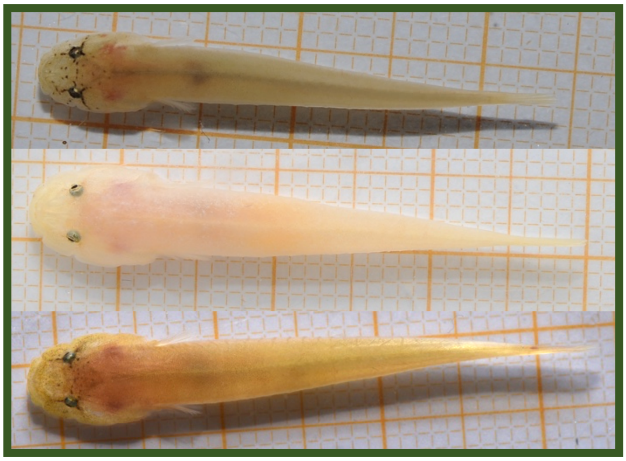

G. adriatica **sp. nov.**
Krk 45.095806, 14.442333;
44.945917, 14.706167

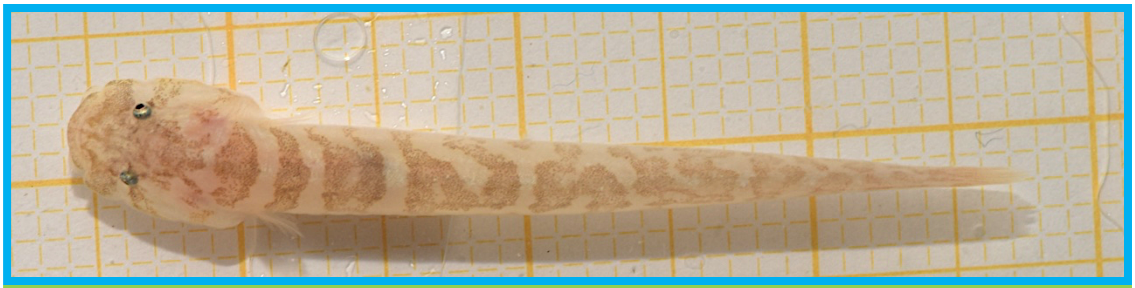

G. hofrichteri **sp. nov.**

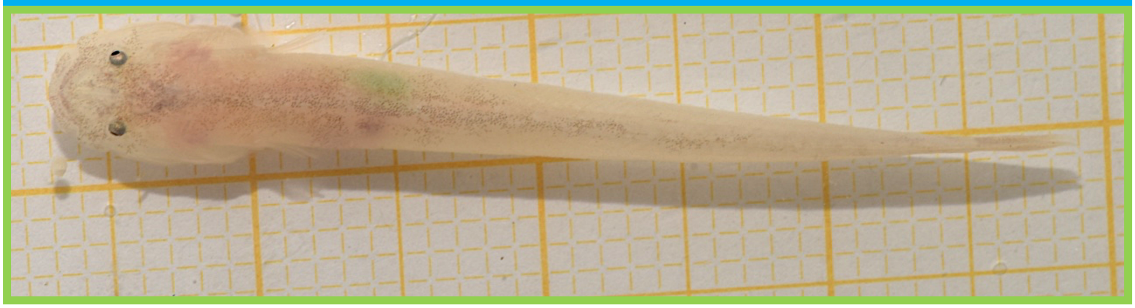

G. pigra (Nardo 1827)

Pelješac 42.902149, 17.429719

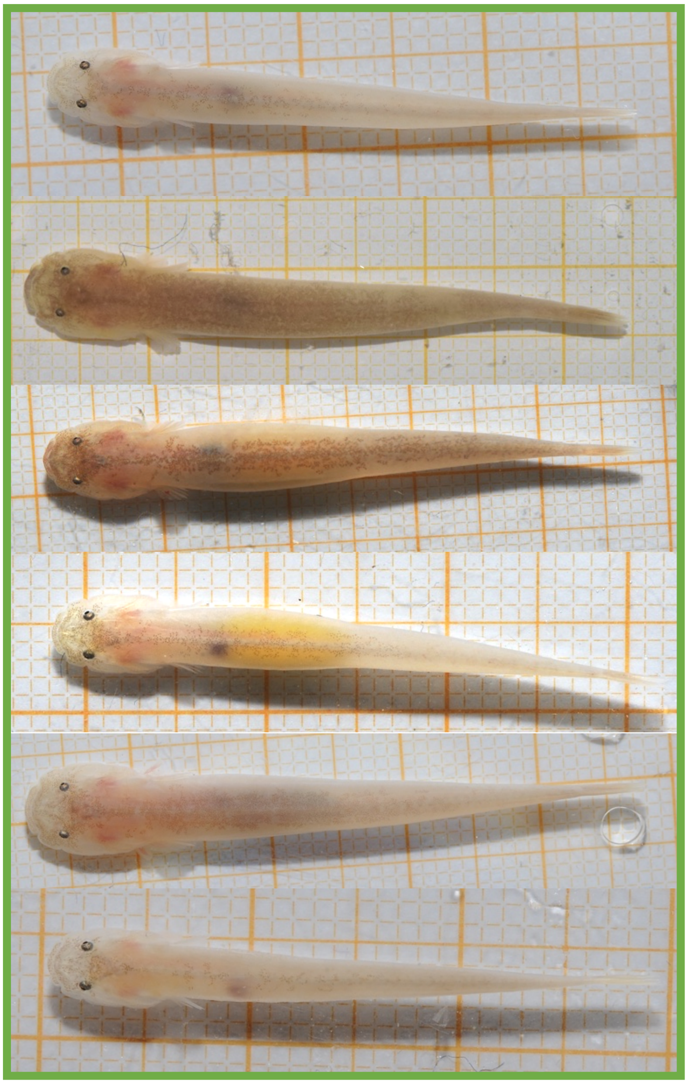

G. pigra (Nardo 1827)
Krk 45.095806, 14.442333; 44.945917, 14.706167

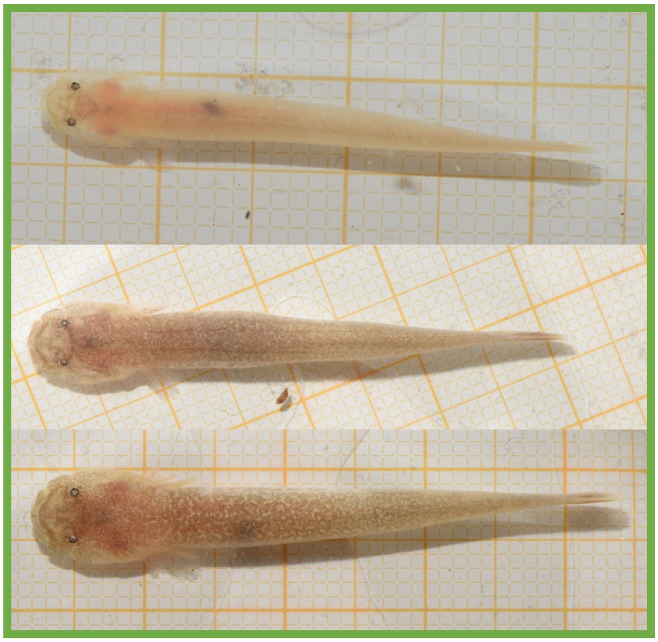

G. pigra (Nardo 1827)
Sv. Marina 45.028361, 14.154833

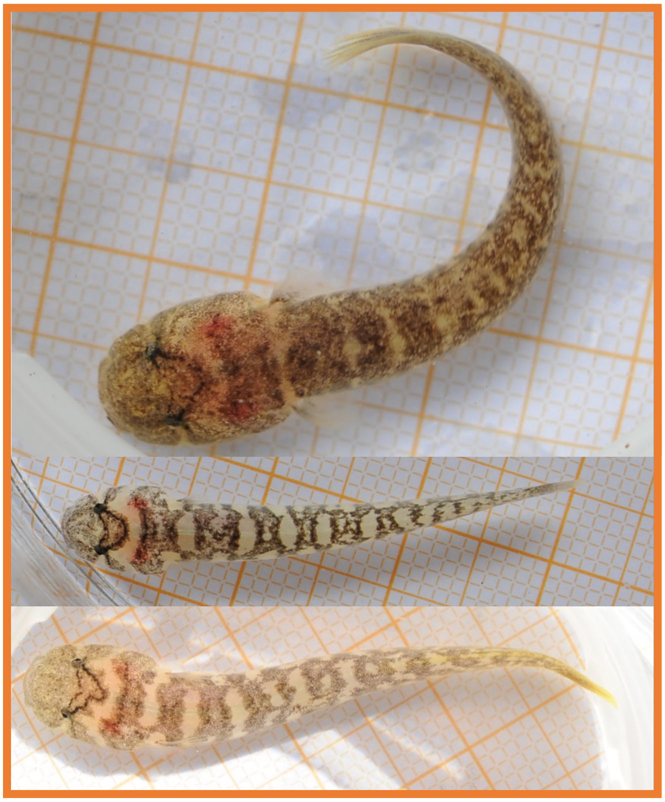

G. willdenowi (Risso 1810)
Messina 38.219137, 15.567788

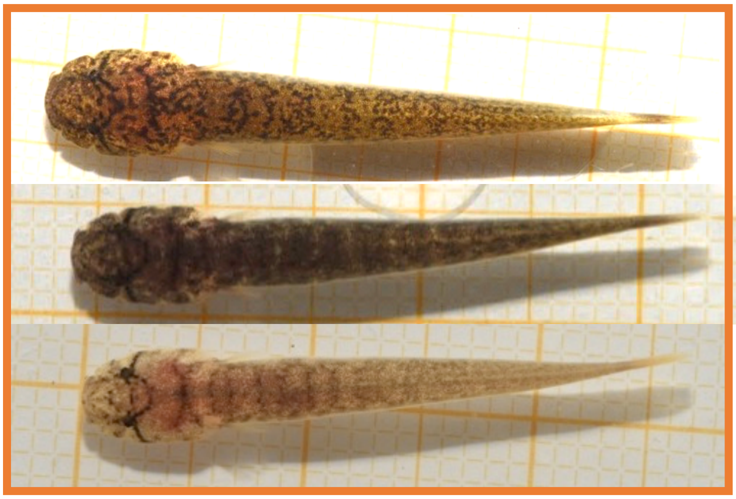

G. willdenowi (Risso 1810)
Banyuls-sur-mer 42.488500, 3.128861

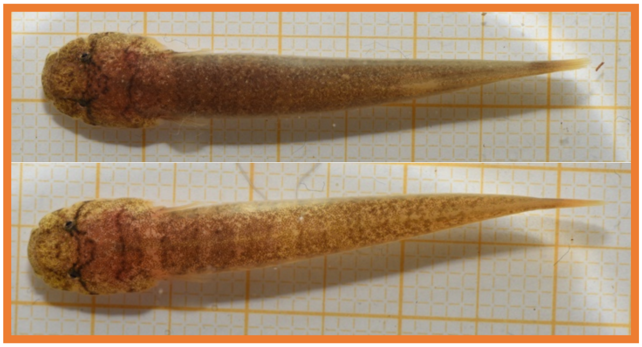

G. willdenowi (Risso 1810)
Toulon 43.146611, 5.707556

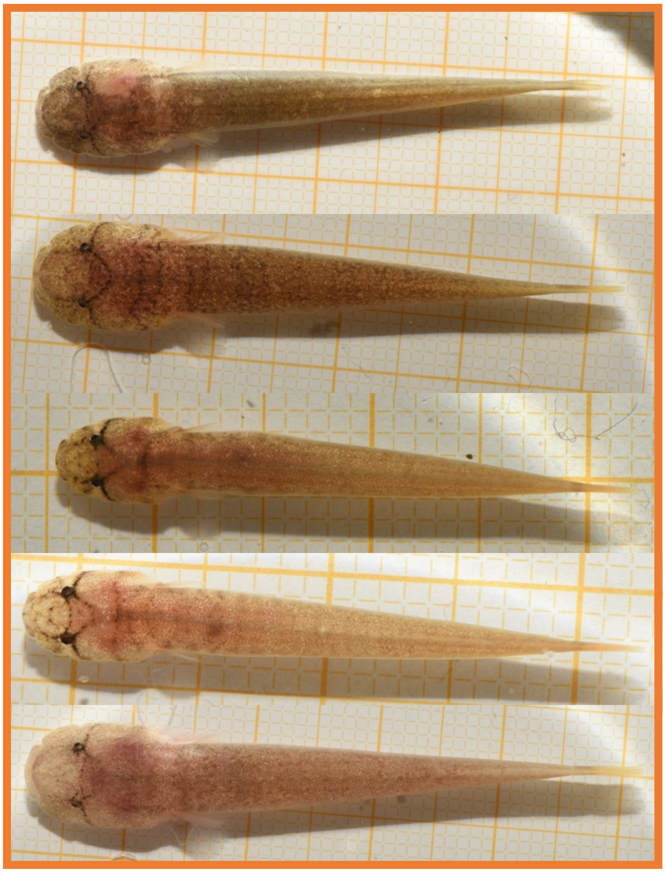

G. willdenowi **(Risso 1810)**
Nice 43.656139, 7.173694 &
43.570056, 7.136528

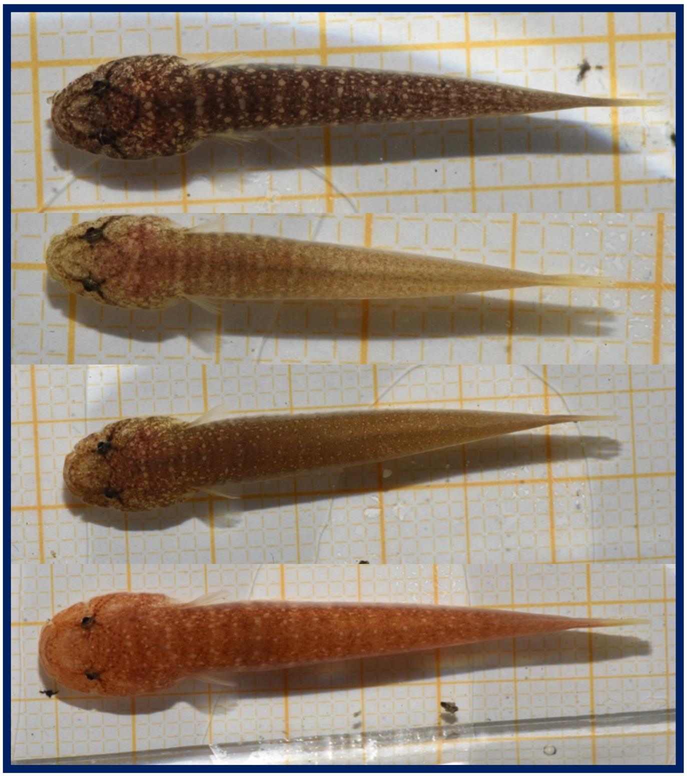

G. orientalis **sp. nov.**
Plakias/Crete
35.194667, 24.380806

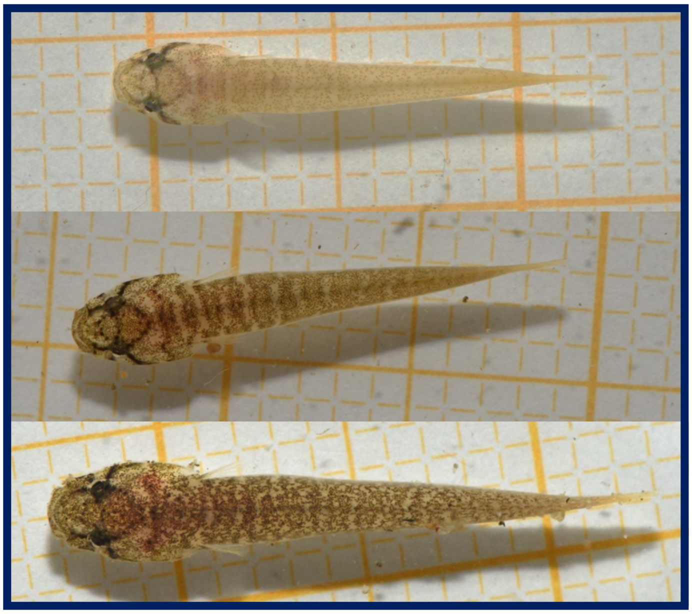

G. orientalis **sp. nov.**
Petres/Crete
35.357833, 24.368972
